# Global small RNA analysis in fast-growing *Arabidopsis thaliana* with elevated concentrations of ATP and sugars

**DOI:** 10.1186/1471-2164-15-116

**Published:** 2014-02-10

**Authors:** Chao Liang, Xuan Liu, Yuzhe Sun, Siu-Ming Yiu, Boon Leong Lim

**Affiliations:** 1 School of Biological Sciences, the University of Hong Kong, Pokfulam, Hong Kong, China; 2 Department of Computer Science, the University of Hong Kong, Pokfulam, Hong Kong, China; 3 State Key Laboratory of Agrobiotechnology, The Chinese University of Hong Kong, Shatin, Hong Kong, China

**Keywords:** Chloroplasts, Mitochondria, miRNAs, MORF, PPR, tasiRNAs and natsiRNAs

## Abstract

**Background:**

In higher eukaryotes, small RNAs play a role in regulating gene expression. Overexpression (OE) lines of *Arabidopsis thaliana* purple acid phosphatase 2 (AtPAP2) were shown to grow faster and exhibit higher ATP and sugar contents. Leaf microarray studies showed that many genes involved in microRNAs (miRNAs) and trans-acting siRNAs (tasiRNAs) biogenesis were significantly changed in the fast-growing lines. In this study, the sRNA profiles of the leaf and the root of 20-day-old plants were sequenced and the impacts of high energy status on sRNA expression were analyzed.

**Results:**

9-13 million reads from each library were mapped to genome. miRNAs, tasiRNAs and natural antisense transcripts-generated small interfering RNAs (natsiRNAs) were identified and compared between libraries. In the leaf of OE lines, 15 known miRNAs increased in abundance and 9 miRNAs decreased in abundance, whereas in the root of OE lines, 2 known miRNAs increased in abundance and 9 miRNAs decreased in abundance. miRNAs with increased abundance in the leaf and root samples of both OE lines (miR158b and miR172a/b) were predicted to target mRNAs coding for Dof zinc finger protein and Apetala 2 (AP2) proteins, respectively. Furthermore, a significant change in the miR173-tasiRNAs-*PPR*/*TPR* network was observed in the leaves of both OE lines.

**Conclusion:**

In this study, the impact of high energy content on the sRNA profiles of Arabidopsis is reported. While the abundance of many stress-induced miRNAs is unaltered, the abundance of some miRNAs related to plant growth and development (miR172 and miR319) is elevated in the fast-growing lines. An induction of miR173-tasiRNAs-*PPR*/*TPR* network was also observed in the OE lines. In contrast, only few cis- and trans-natsiRNAs are altered in the fast-growing lines.

## Background

**Figure 1:**
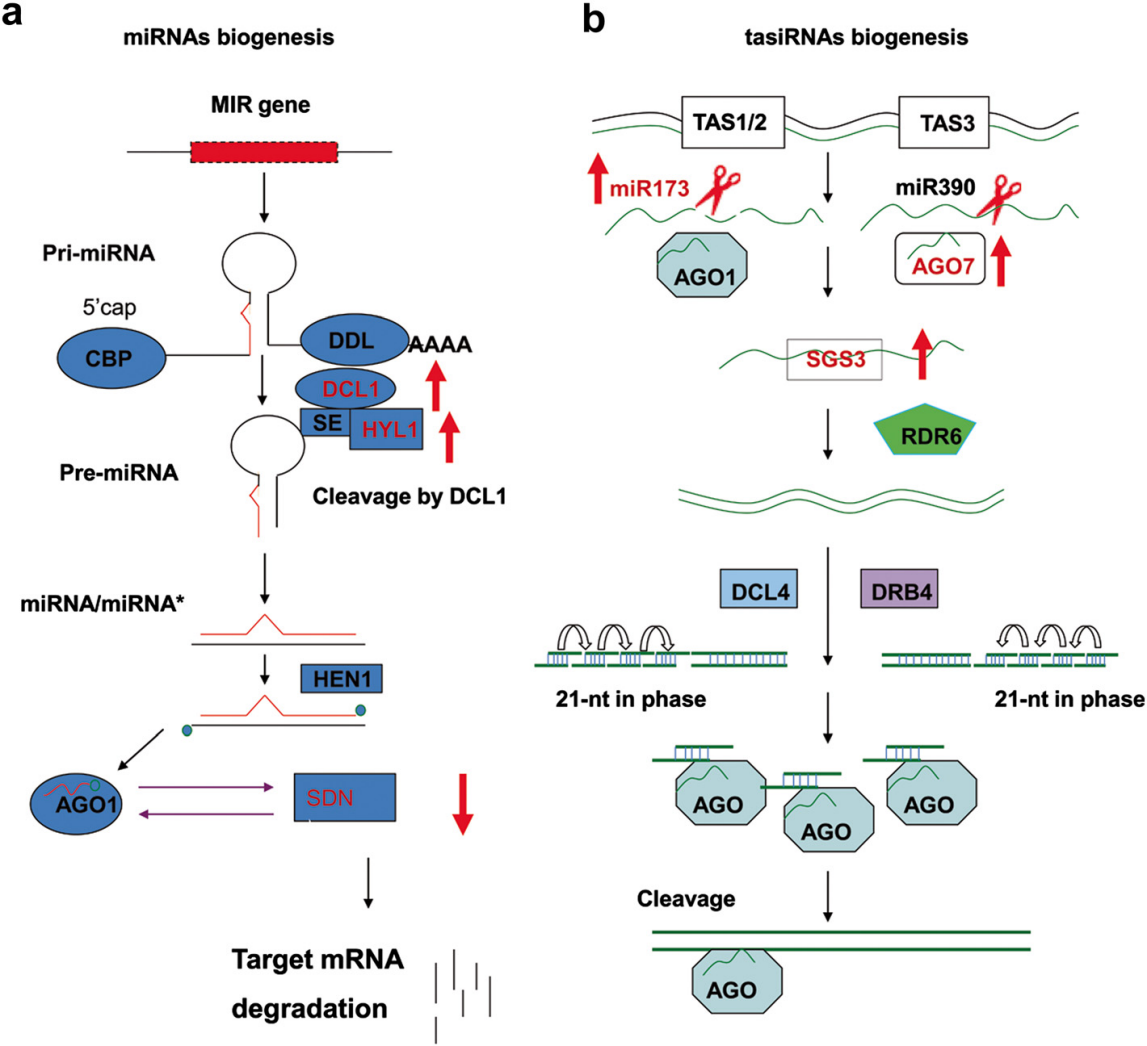
**Significant changes of leaf microarray data in genes for miRNAs (a) and tasiRNAs (b) biogenesis.** The red arrows indicate genes with significantly altered transcript abundance in OE leaf.

Small RNA silencing is an essential mechanism in gene regulation in eukaryotes. There are two main categories of small RNAs: microRNAs (miRNAs) and small interference RNAs (siRNAs), which are generated from single-stranded, self-complementary RNA transcripts and double-stranded RNAs (dsRNAs), respectively. The primary transcript of a *MIR* gene is called pri-miRNA, which is further processed into the stem-loop precursor miRNA (pre-miRNA) by Dicer like 1 (DCL1). While the guide strands of the miRNA duplexes are incorporated into ARGONAUTE 1(AGO1) of the RNA-induced silencing complex (RISC), the passenger strands called miRNA star (miRNA*) are mostly degraded (Figure 1) [1].

The sources of dsRNAs that trigger siRNAs biogenesis could be exogenous (e.g., viral replication) or endogenous. Plant evolved several classes of endogenous siRNAs including tasiRNAs, natsiRNAs and cis-acting siRNAs (casiRNAs). In plants, tasiRNAs are generated by a pathway different from that of miRNAs (Figure 1). The genomic loci encoding tasiRNAs are known as *TAS* genes and are transcribed by polII. The generation of tasiRNA is initiated by miRNA-mediated cleavage of long non-coding transcripts of *TAS* genes. Eight *TAS loci* from four families (*TAS1-4*) are identified in *Arabidopsis* genome [2-4]. There are three loci in TAS1 family, *TAS1A* (At2g27400), *TAS1B* (At1g50055) and *TAS1C* (At2g39675). Both *TAS1* and *TAS2* (At2g39681) transcripts are cleaved by miR173 and associated with AGO1 to generate siRNAs, which mainly target pentatricopeptide repeat-containing (PPR) mRNAs [5-7]. There are three *TAS3* loci in *Arabidopsis thaliana*, *TAS3A* (At3g17185), *TAS3B* (At5g49615) and *TAS3C* (At5g57735). miR390 guides cleavage of these transcripts with AGO7 to generate siRNAs which target mRNAs of auxin responsive factors (ARF) family (e.g. ARF2, ARF3 and ARF4) [5,8,9]. *TAS4* transcript is initiated by miR828 in association with AGO1 to generate tasiRNAs and their targets are MYB transcription factors [10]. The cleaved RNAs from the eight loci are bounded by suppressor of gene silencing 3(SGS3) and copied into dsRNAs by RNA-dependent RNA polymerase 6 (RDR6). The dsRNAs are cleaved in multiple rounds by DCL4 from the end defined by miRNA-mediated cleavage such that the tasiRNAs are in 21-nucleotide (nt) register from the cleavage site. The tasiRNAs are loaded into AGO1 complex to initiate tasiRNA guided mRNA degradation [4,11]. Another class of siRNAs is nat-siRNAs, which could be derived from RNAs transcribed from opposite strands of the same loci (cis-nat-siRNAs) [12] or by transcripts from different loci (trans-nat-siRNAs). There are 1,739 and 4,828 potential cis- and trans- natural antisense transcripts (NATs), respectively in *Arabidopsis*[13]. The production of nat-siRNAs are is dependent on RDR6 and DCL2 (24-nt) or DCL1 (21-nt).

Overexpression of *AtPAP2* in *A. thaliana* speeds up plant growth. The OE lines flower early and grow faster than the wild type (WT) plants. The seed yield and silique numbers of OE lines are also more than the control lines [14]. AtPAP2 was shown to be dually targeted to chloroplasts and mitochondria [15]. Metabolomics studies showed that some sugars (sucrose, glucose, fructose and myo-inositol), TCA metabolites (citrate, fumarate, malate and succinate) and certain amino acids (alanine, glycine, glutamate, proline, serine and valine) are significantly higher in the OE lines than in the WT [14]. The concentrations of ATP and ADP are also higher in the OE lines [16]. All of these phenotypes pointed to a dramatic shift of metabolism in the overexpression lines [14]. Besides, overexpression of *AtPAP2* in another member of Brassicaceae (*Camelina sativa*) can also generate fast growing and higher seed-yield transgenic plants [17].

Microarray data showed that thousands of transcripts were significantly altered (P < 0.05) in the OE lines [16], including key genes involved in carbon flow, K uptake and nitrogen assimilation. In addition, the transcription of many genes involved in miRNAs and siRNAs biogenesis was altered (Table 1). The transcript abundance of key genes involved in miRNA biogenesis, including *DCL1* and *HYL1*, significantly increased (P < 0.05) in both OE lines, whereas *SDN1*, responsible for miRNA degradation, had transcript abundance decreased in both OE lines (Figure 1). The transcript abundance of essential genes involved in tasiRNA biogenesis, including *SGS3*, *AGO1* and *AGO7*, also significantly increased (P < 0.05) in both OE lines. To examine the small RNAs profiles in the fast-growing lines, we constructed eight sRNA libraries from both leaves and roots of wild type (WT), *pap2* (mutant in the same background), and two independent overexpression lines (OE7 and OE21). This study provides information on the impacts of high energy status (ATP, sugars) on small RNA profiles in *Arabidopsis*.

**Table 1:** **Microarray data of genes involved in miRNA and siRNA biogenesis in WT, *pap2*, OE7 and OE21**

**Gene**	**WT**	***pap2***	**OE7**	**OE21**	**OE7 FC**	**OE21 FC**	**P OE7**	**P OE21**	**Primary gene symbol**
AT1G48410	9057	9424	10052	10717	111%	119%	0.322	0.057	Argonaute 1 (AGO1)
AT2G27880	225	218	415	354	183%	154%	0.037	0.048	Argonaute 5 (AGO5)*
AT1G69440	1073	853	2149	2357	200%	219%	0.002	0.000	Argonaute 7 (AGO7)*
AT5G44200	3599	3013	4053	4057	113%	113%	0.234	0.299	CPB20
AT2G13540	6824	6887	6310	6274	92%	92%	0.415	0.440	CPB80
AT1G01040	1543	1308	2319	2501	150%	162%	0.016	0.006	Dicer-like 1 (DCL1)*
AT3G43920	2701	2633	1984	2042	74%	75%	0.046	0.029	Dicer-like 3 (DCL3)*
AT4G20910	1308	1257	1139	1403	87%	107%	0.364	0.619	HEN1
AT1G09700	2576	2610	3368	3881	130%	150%	0.049	0.005	Hyponastic leaves 1 (HYL1)*
AT1G14790	1673	1576	2528	2874	149%	170%	0.052	0.009	RNA-dependent rna polymerase 1 (RDR1)*
AT5G23570	2079	2333	5530	6674	265%	322%	0.011	0.000	Suppressor of gene silencing 3 (SGS3)*
AT3G50100	1588	1368	446	647	28%	41%	0.004	0.000	Small RNA degrading nuclease 1 (SDN1)*

*Significantly (p < 0.05) changed in both OE lines. FC: Fold change.

The following genes were not significantly (p > 0.05) different between the WT and OE lines:

AGO2, AGO4, AGO6, DCL2, DCL4, HEN 1, HEN2, HEN4, HASTY, NRPD1A, RDR2, RDR6 (SGS2), SDE3 and SDE5.

## Results

### Small RNAs length distribution and annotation

Eight small RNA libraries were generated from the leaves and the roots of 20-day-old *Arabidopsis*. After removal of adaptors and low quality tags, the number of cleans reads were calculated and mapped to the *A. thaliana* genome. In total, 13 to 19 million raw reads were generated from each library, and approximately 95% of the reads remained for further research after clearing the adaptors sequences. Approximately 9 to 13 million sequence reads (approximate 73 - 87% of the clean reads) corresponding to 0.5 to 1.3 million unique sequence signatures could be mapped onto the genome (Additional file 1).

The length of the clean reads ranged from 18 to 30 nucleotides. The size distribution of small RNA sequences in various leaf (a) and root (b) samples were reported in Additional file 2. Similar distributions were observed in OE, *pap2* and wild type leaf libraries. Consistent with the earlier reports [18], two major peaks appeared at 21 and 24 nucleotides in length in the total sequence reads (Additional file 2a). In the root libraries, an additional peak at 19 nucleotides was observed. These 19-nucletide sequences are mainly originated from tRNAs, and they are cleaved from the 5′ end of Gly-tRNA^TCC^ which represented over 80% of tRNA-derived small RNAs in the roots [18]. Interestingly, the total reads of these 19-nucletide sequences are relatively more (approximate 6 million) in both OE lines than in WT (3.8 million) and *pap2* (3.4 million) (Additional file 2b). Clean reads from the eight libraries were mapped and classified according to the annotation of the genome. A significant higher proportion of small RNAs were mapped to tRNA genes in the root libraries (21.28 - 47.30% of the clean reads) than in the leaf libraries (3.60 - 6.96%). For the repeat small RNAs, both unique and total reads are less in OE leaf and root samples. We observed that the total reads of sRNA antisense to exons (Exon antisense) are higher in leaves of the OE lines, while there are smaller number of unique reads (Additional file 3); these indicate that the sRNA antisense to exons are more concentrated to their targets in the leaves of OE lines.

### Identification of known miRNAs

**Figure 2:**
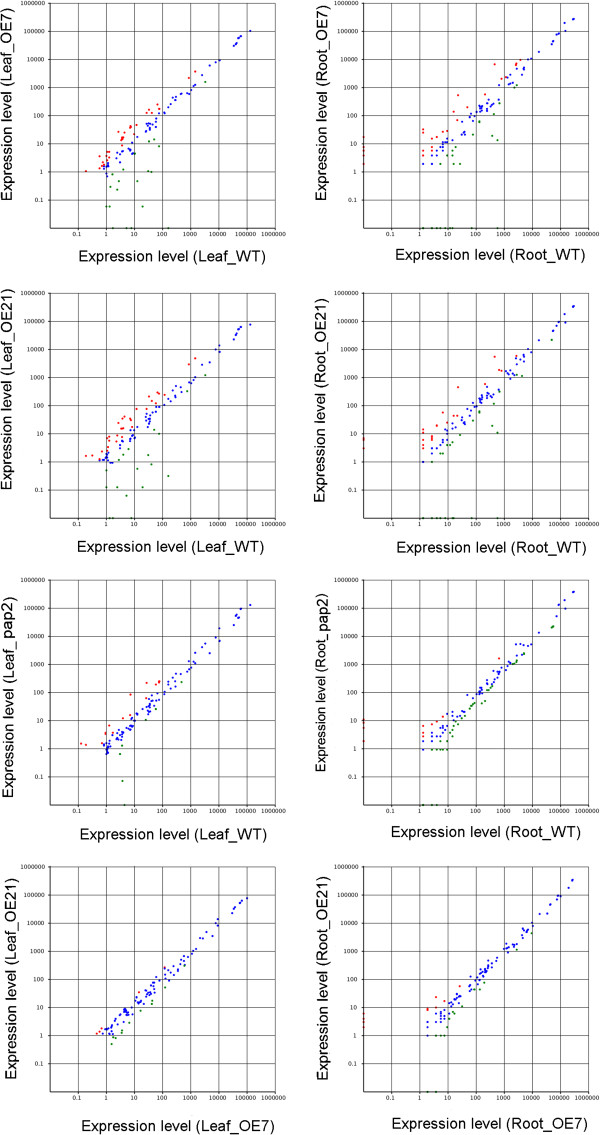
**Scatter plot of known miRNAs (Leaf and root).** Red dots: Increase in abundance (ratio > 2, P < 0.05); blue dots: Equal expression (1/2 < ratio ≤2); Green dots: Decrease in abundance (ratio ≤ 1/2, P < 0.05). In all the presented figures, X-axis indicates control while Y-axis indicates treatment sample.

It has been reported that high throughput sequencing can be an alternative to estimate the expression profiles of miRNA genes [19]. This method allows us to distinguish the transcript abundance of the same miRNA gene between different lines. Small RNAs were mapped to the miRNA precursor/mature miRNA species in miRBase15.0 (http://www.mirbase.org/). Expression profiles of all known miRNAs of the eight libraries were listed including 243 miRNAs (Additional file 4). Scatter plot (Figure 2) showed the differential expression of miRNA between different lines. The miRNAs expression patterns between the two OE lines are indifferent, while comparing to WT as controls, there are significant changes.

Table 2 shows the miRNAs with significant differential expression between the OE lines and the WT. All the known miRNAs were calculated by fold change log2 (OE/WT) >1 or < -1 and p-value <0.05. The miRNAs significantly changed in the OE lines mainly target to the mRNAs of several protein families including SPL family, HAP2-like transcription factors, Apetala 2-like transcription factors, TCP transcription factors, TAS family, laccases, cation/hydrogen exchangers and jacalin lectins. These miRNA expression data was cross-examined with the leaf microarray data. Increases in some miRNAs abundance correlates with the decreases in mRNA transcript abundance, including ath-miR172a,b, ath-miR172c, ath-miR319a,b and ath-miR846. The targets are Apetala2-like transcription factors (Licausi et al., 2013), TCP transcription factors and jacalin lectins, respectively. The abundance of miR173, well known to be responsible for initiating tasiRNA biogenesis for *TAS1A*, *TAS1B*, *TAS1C* and *TAS2*, significantly increased in the leaves of both OE lines. In contrast, the abundance of miR391, a member of the miR390 family [20], significantly decreased in the leaves of both OE lines.

**Table 2:** Known miRNAs with significant changes between both OE lines and WT

**miRNAs**	**Root (counts)**				**Leaf (counts)**				**Target gene family**
	**OE21**	**OE7**	***pap2***	**WT**	**OE21**	**OE7**	***pap2***	**WT**	
Increase in abundance in OE vs WT									
ath-miR158b	**4864**	**3394**	305	409	**3101**	**2382**	858	494	PPR(At1g64100)
ath-miR172a,b	1493	1204	649	1019	**70458**	**54544**	38556	21624	Apetala 2-like transcription factors
ath-miR172c	39	46	28	54	**1123**	**691**	267	178	Apetala 2-like transcription factors
ath-miR173	4900	2619	2405	4614	**43178**	**32463**	19271	13142	TAS1A, TAS1B, TAS1C, TAS2
ath-miR319a,b	5	9	9	0	**232**	**252**	57	61	TCP Transcription factor
ath-miR397a	39	71	7	14	**449**	**590**	99	114	Laccase
ath-miR780.1	0	0	5	7	**597**	**376**	**0**	67	Cation/hydrogen exchanger
ath-miR780.2	0	0	0	4	**516**	**218**	**1**	57	Cation/hydrogen exchanger
ath-miR835-3p	5	0	0	1	**103**	**77**	16	16	LYR family of Fe/S cluster biogenesis protein
ath-miR839	**410**	**271**	8	20	**364**	**392**	32	40	Unknown target
ath-miR841	13	13	0	1	**2161**	**1874**	1176	632	Unknown target
ath-miR841b*	40	35	14	19	**4334**	**3674**	1369	996	Histone protein
ath-miR842	1164	661	1293	1288	**114**	**248**	39	55	Jacalin lectin
ath-miR846	235	189	128	256	**512**	**545**	**232**	107	Jacalin lectin
ath-miR851-3p	51	14	4	6	**227**	**200**	31	51	Unknown target
Decrease in abundance in OE vs WT									
ath-miR157a,b,c	**19563**	**18016**	**21655**	43815	935537	1004505	1408657	921290	Squamosa-promoter binding protein-like
ath-miR169h,k,m	**50**	**30**	108	120	125	66	91	125	HAP2-like transcription factors
ath-miR169i,j,l,n	**279**	**140**	570	611	344	180	305	490	HAP2-like transcription factors
ath-miR3440b-3p	**104**	**58**	243	385	**207**	**210**	503	753	Unknown function
ath-miR391	223	**135**	609	373	**17772**	**23755**	80936	50073	TAS3
ath-miR396a	**1124**	**613**	1443	2513	**4902**	9371	8279	11425	Growth Regulating Factor
ath-miR397b	0	0	35	62	**2**	**1**	381	297	Laccase
ath-miR773	**17**	**10**	176	336	**26**	**16**	252	474	DNA(cytosine-5-)-methyltransferase
ath-miR775	**0**	**0**	355	521	**5**	**0**	2876	2418	Galactosyltransferase
ath-miR822	**1026**	**1437**	2301	3882	7423	6893	6903	4528	Cysteine/Histidine-rich C1 domain family protein
ath-miR833-5p	0	0	21	13	**0**	**0**	171	119	Unknown target
ath-miR843	1	1	0	1	**149**	**119**	803	1136	F-box family protein
ath-miR848	**10**	**7**	567	518	**12**	**15**	732	617	Unknown target
ath-miR857	0	0	6	5	**8**	**7**	262	195	Laccase

Numbers in bold mean significant changes in OE versus WT, p-value <0.05 and fold change log2 (OE/WT) >1 or fold change log2 (OE/WT) < -1. All reads were normalized. The reads cut off was 100. All the targets were predicted from website of Mayer’s lab (http://mpss.udel.edu/at_sRNA/index.php?menu = https://wasabi.dbi.udel.edu/~apps/tp/index.php?SITE = at_sRNA).

### Analysis of novel miRNAs candidates and their targets

Novel miRNAs and their target genes were predicted by the Mireap software. Target genes are predicted based on the rules suggested by Allen et al. [5] and Schwab [21]. Secondary structures were predicted and analyzed for stable stem-loop hairpins (Additional file 5). The detail of novel *MIR* genes including location, minus free energy (MFE), sequence and structures are listed in Additional file 6. According to the criteria described in methods, seven novel miRNAs with counts > 100 were identified in leaf libraries, of which two were also identified in root libraries (Additional file 7). For leaf/root_miRNA0001RNA_5p, it was predicted to target to chromomethylase1 (CMT1). And for leaf_miRNA0002_5p and root_miRNA0002_3p, only found in WT/*pap2*, were predicted to target retrotransposon ORF-1 protein. Leaf_miRNA0003_3p and leaf_miRNA008_5p, which had relatively high reads, were only found in *pap2* (3281 reads) and WT (2001 reads), respectively and their targets were six F-box family proteins. Leaf_miRNA0005_5p and leaf_miRNA0006_3p were predicted to target pentatricopeptide repeat (PPR) family proteins (AT4G28010 and AT1G62260), both are proteins located in mitochondria. These two miRNAs were either not found (leaf_miRNA0006_3p) or the counts were too low (leaf_miRNA0005_5p) in the leaves of WT and *pap2* line and therefore not detected in previous studies.

### Trans-acting small interference sRNAs analysis

**Figure 3:**
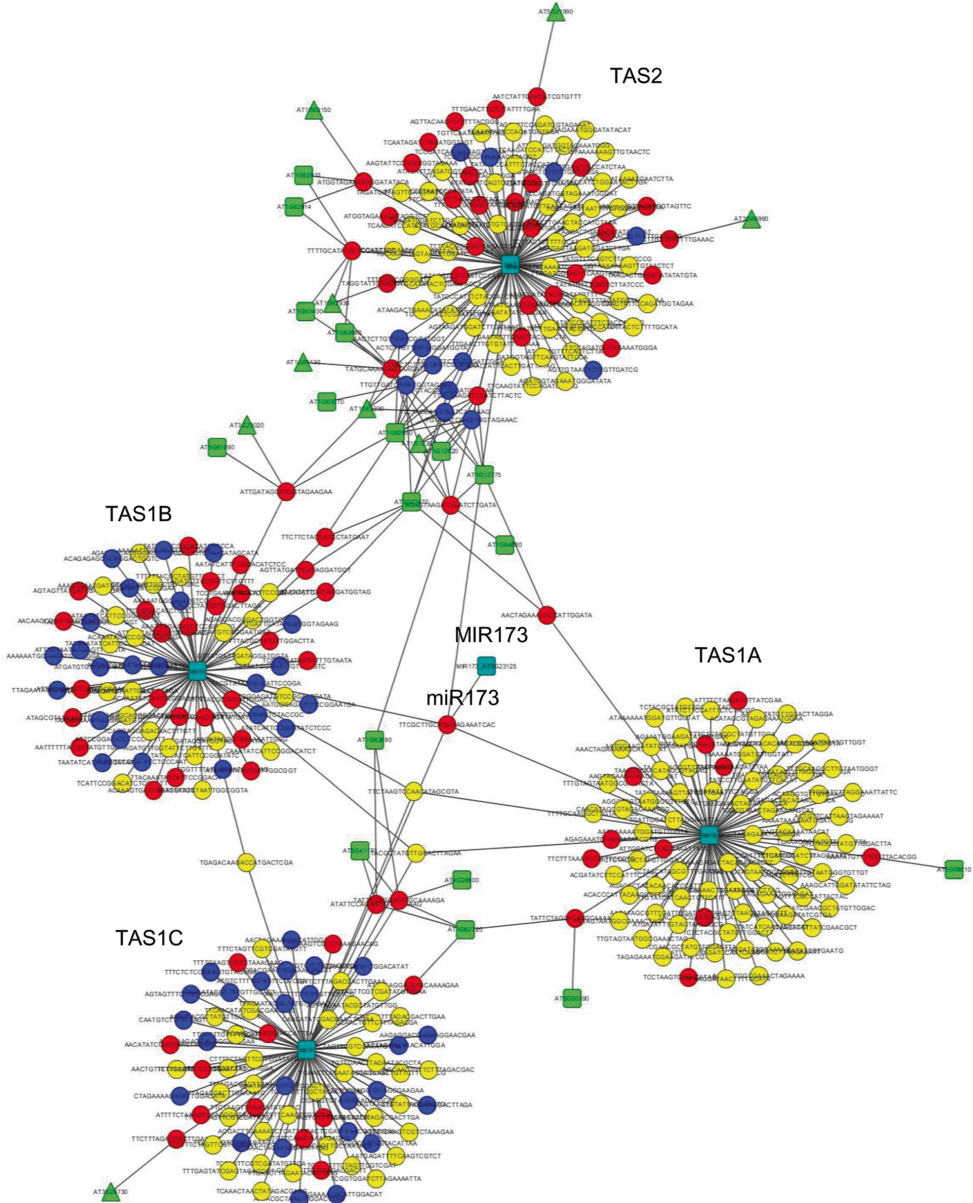
**The miR173-tasiRNAs-*PPR*/*TPR *network is significantly altered in the OE lines.** Circles are 21-nt small RNAs that are perfectly mapped to *TAS* loci; red circles: Increase in small RNAs abundance in both OE lines (ratio ≥ 2, P < 0.05); blue circles: Decrease in small RNAs abundance in both OE lines (0 < ratio ≤ 1/2, P < 0.05); yellow circles: small RNAs which are not significantly changed in the OE lines. Green squares: potential *PPR* targets. Green triangles: potential *TPR* targets. Dark green squares: *TAS1A*, *1B*, *1C*, *TAS2* and *MIR 173* genes.

The mapping of all the clean reads to the eight tasiRNAs loci, 21-nt tasiRNA reads were normalized and compared between samples. There were significant changes in the tasiRNA profiles in the leaves of both OE lines (Additional file 8). As stated above, the counts of miR173 were significantly higher in the leaves of both OE lines. miR173 was known to induce cleavage of *TAS1* and *TAS2* transcripts and initiate tasiRNAs production from these loci (Chen et al., 2007). Our data shows that the amounts of tasiRNAs generated from *TAS1A, B, C* and *TAS2* were significantly increased in the leaves of the OE lines. Phase register and count distribution of *TAS1A, TAS1B, TAS1C and TAS2* sites cleaved by miR173 in leaf were presented (Additional file 9). The phase distribution pattern of *TAS1B* was significantly altered between OE and WT lines (Additional file 9). The predicted targets of many of these tasiRNA are mRNAs of PPR and TPR genes. In the four leaf samples, 360 individual 21-nt sRNA sequences with reads >10 could be mapped to the four *TAS1* and *TAS2* loci, of which 87 sRNA sequences were found to be significantly differentially expressed between the WT and both OE lines (Additional file 8). The same sRNA sequences could be generated from more than one locus, and multiple sRNAs are predicted to target to the same target. A network of miR173-tasiRNAs-*PPR*/*TPR* was generated to show their relationship (Figure 3). All the potential PPR/TPR genes that appeared in the network were listed in Additional file 10. For *TAS4*, the abundance of tasiRNA TAS4-SiR81 (-) (5′-TGAAGGATCGAGGTCGAGGCA-3′) strongly decreased in both OE lines (1,728 and 3,698 reads) compared to the WT (13,783 reads). In contrast, there are no significant changes in the tasiRNAs generated from the three *TAS3* loci (Additional file 8).

### Natural-antisense-transcript derived small interference RNAs

Out of 1,739 potential cis-NATs predicted from *Arabidopsis* TAIR 10 database, we could only map our sRNAs to 1665 cis-NATs; only 44 (2.6%) and 36 (2.1%) pairs were significantly altered in the leaves and roots of both OE lines (Additional file 11). Only 12 cis-NATs increased in abundance in the leaves of the OE lines, some have significant fold changes. The genes in the gene pairs include 3 miRNAs (miR780, miR836, miR841), *TAS1C*, one *PPR* gene, two genes involved in auxin response, a histone *H2A* gene and a phosphoribosylanthranilate isomerase gene. The sRNA counts for the latter two gene pairs increased by 10- and 60-fold in leaves, respectively. Some cis-NATs strongly decreased in abundance in the leaves of OE lines. The genes in the gene pairs include genes of Lhcb1.5, Lhcb6, a protein kinase APk2b, 2 F-box proteins and a UDP-3-O-[3-hydroxymyristoyl] N-acetylglucosamine deacetylase. The sRNA mapped to the latter gene pair dropped ~100-fold in both leaves and roots.

Out of 4,828 possible trans-NATs pairs, only 253 (5.2%) and 256 (5.3%) pairs were significantly altered in the leaves and roots of both OE lines, respectively (Additional file 12). Most of the genes involved are transponsable elements (264 and 302 genes in the leaf and root datasets), pseudogenes (38 and 10 genes in leaf and root datasets) and genes for unknown proteins (87 and 86 genes in leaf and root datasets).

### Validation of siRNAs and candidate genes by qRT-PCR

To validate the sRNA sequencing data, the abundance of selected miRNAs (miR173, miR390 and miR391) and 21-nt tasi-RNAs mapped to CDS of *TASIB* (551-571), *TAS1C* (378 – 398), *TAS2* (629 – 649) and *TAS4*-siR81(-) in different lines were compared by qRT-PCR. All the results of selected small RNAs were consistent between qRT-PCR and sequencing. The microarray data has been validated by qRT-PCR in our previous study [16] and the transcript abundance of selected genes, including *SGS3*, *At1g12620* (PPR), were also well validated by qRT-PCR in this study (Additional file 13).

## Discussion

sRNA biogenesis is one of the regulatory mechanisms in organism. In this study, the sRNA profiles of high energy plants were compared with that of WT plants under soil-grown condition. The size and growth stages of the plants were identical so that variations due to developmental stages and morphology were minimized. Many miRNAs and siRNAs were activated by various biotic and abiotic stresses and subsequently modulated the mRNA stability of their target genes, so that the plants can cope with the stresses accordingly [22]. Our data shows that very few miRNAs were significantly altered in the OE lines under the experimental conditions. Out of 243 known miRNAs in *Arabidopsis*, only 15 and 9 miRNAs significantly increased or decreased in abundance in the leaves of OE lines. Very few miRNAs were altered in the roots, only 2 and 9 miRNAs significantly increased or decreased in abundance, respectively.

Some miRNAs were shown to control plant development [23]. Overexpression of miR156 and miR159 resulted in late-flowering [21,24], and overexpression of miR160 resulted in increased lateral rooting [25]. However, the abundance of these miRNAs was unaltered in the OE lines. Only the abundance of miR172 and miR319 were significantly higher in the leaves of the OE lines (Table 2). The targets of miR172 and miR319 are AP2 and TCP, transcription factors, respectively. Both are involved in leaf development, floral organ identity and flowering time [21,26-28]. Overexpression of miR172 in *Arabidopsis* and potato can promote flowering development and tuberization respectively [29]. The higher expression of miR172 in the leaves of OE lines correlates with the earlier bolting and flowering phenotypes of the OE lines [14]. For miRNAs that are induced by nutritional stresses, such as phosphate deficiency (miR399) copper deficiency (miR398), and sulfate deficiency (miR395), their abundance were low in all lines and were not significantly changed in our OE lines [22,30-32]. This is reasonable as the plants were grown in soil with adequate supply of nutrients. Similarly, miRNAs responsive to bacterial (miR160, miR167, miR393, miR396, miR398 and miR825) and viral infections (miR156 and miR164) were not altered in the OE lines [33-35]. The abundance of some miRNA (miR172 and miR397) induced by cold stresses increased in the OE lines but many other miRNAs (miR166, miR393, miR396 and miR408) induced by cold were unaltered [36]. Accumulation of sucrose in the cytosol is a key protection mechanism for cold tolerance. Since the OE lines contain higher sucrose contents [14], miR172 and miR397 might be induced indirectly by cold-induced sucrose accumulation.

Currently, eight *TAS* genes of tasiRNAs have been identified in the *Arabidopsis* genome. The transcribed mRNAs from these loci require miRNA-induced cleavage to generate functional tasiRNAs, which in turn induce the cleavage of the target mRNAs. For example, the *TAS1A/B/C* and *TAS2* tasiRNA families are induced by miR173, which then down-regulate mRNAs of various *PPR* and *TPR* genes [37].Our data shows that the miR173-tasiRNAs-*PPR*/*TPR* network had substantial changes in the leaves of both OE lines (Figure 3). A *TAS* gene prediction algorithm predicted seven *TAS* loci in *A. thaliana* (*P* < 0.0006) using Col-0 MPSS small RNA data [37], which include *TAS1A*/*B*/*C*, *TAS2*, and 3 *PPR* genes (At1g63070, At1g63080 and At1g63130). sRNA generated from these three protein-coding, *PPR* genes were predicted to target to the mRNAs of a number of P-class *PPR* genes [37]. The gene expression signals of these three potential tasiRNA generating *PPR* genes (AT1G63070, FC ≤ 0.21; AT1G63080, FC ≤ 0.63; AT1G63150, FC ≤ 0.46) were indeed suppressed in the leaves of both OE lines in microarray studies (Table 3). There are 441 *PPR* genes in the *Arabidopsis* genome [38]. The transcript abundance of 52 and 25 *PPR* genes were significantly (P < 0.05, FC ≥ ± 1.3) changed in the leaves and the roots of both OE lines (*vs*. WT) (Additional file 14). The miR173-tasiRNAs-*PPR*/*TPR* network should play a role in the regulation of a specific group of *PPR* genes.

**Table 3:** **Microarray data of *PPR *** **genes that had significant changes between both two OE lines and wild-type**

**AGI code**	**WT**	***pap2***	**OE7**	**OE21**	**OE7 FC**	**OE21 FC**	**P OE7**	**P OE21**	**Gene name**
AT1G12620	2259	2920	231	207	10%	9%	0.001	0.000	PPR gene
AT1G62590	188	140	78	86	44%	48%	0.073	0.045	PPR gene
AT1G62910	716	906	1151	1200	162%	169%	0.026	0.002	PPR gene
AT1G63070	666	998	99	138	15%	21%	0.001	0.000	PPR gene
AT1G63080	1143	1336	551	716	48%	63%	0.004	0.004	Transacting siRNA generating PPR gene
AT1G63130	1068	1113	752	740	69%	68%	0.070	0.011	Transacting siRNA generating PPR gene
AT1G63150	1147	1050	350	522	31%	45%	0.007	0.001	Transacting siRNA generating PPR gene
AT1G63230	541	486	223	228	41%	42%	0.001	0.000	PPR gene
AT1G63320	441	580	798	988	204%	247%	0.029	0.030	PPR gene
AT1G63330	2070	1836	700	885	33%	43%	0.004	0.001	PPR gene
AT1G63400	1312	1479	850	869	64%	65%	0.048	0.011	PPR gene
AT1G63630	218	208	686	754	311%	349%	0.043	0.001	PPR gene
AT1G64100	419	507	920	985	221%	237%	0.005	0.000	PPR gene

FC: Fold change.

**Figure 4:**
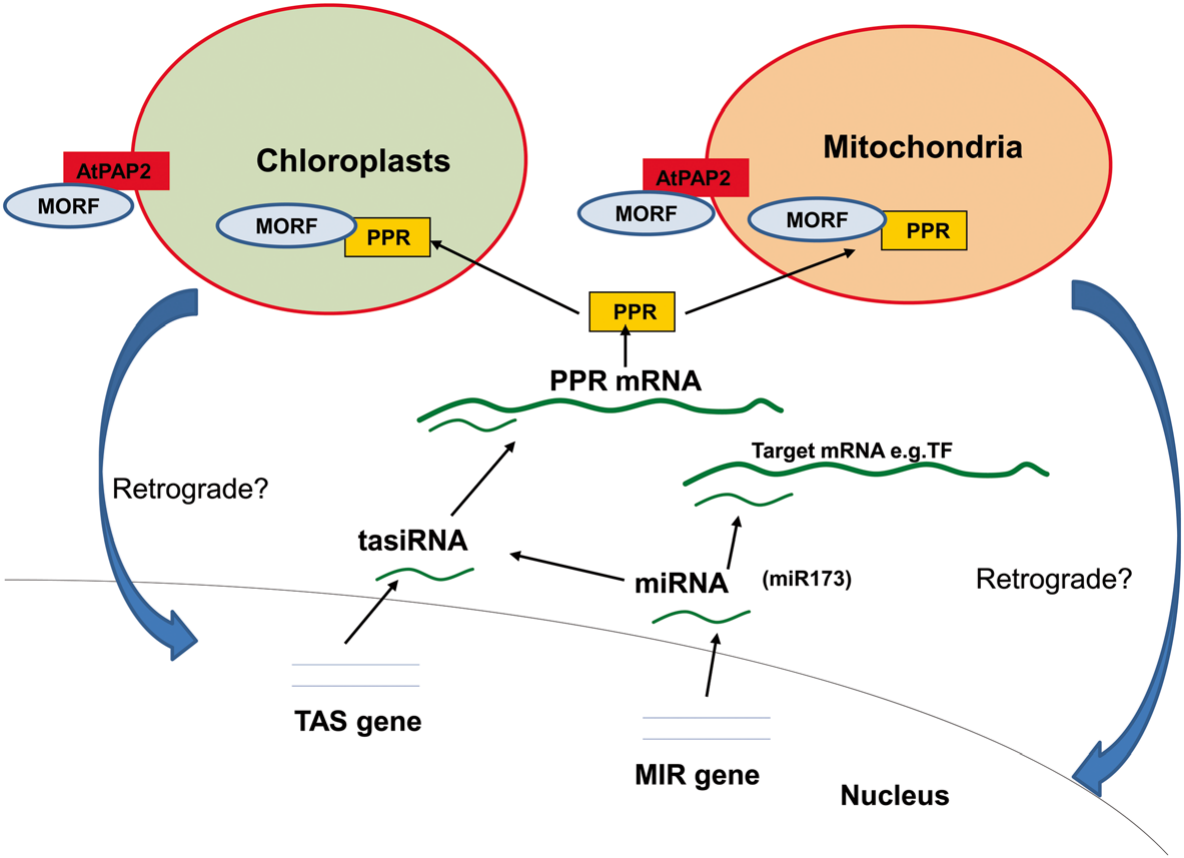
**A model on the relationship of AtPAP2 and sRNAs.** Overexpression of AtPAP2 protein in chloroplasts and mitochondria affect the physiology of these two energy-generating organelles, which may lead to a change in the *miR*173-tasiRNAs-PPR/TPR network.

AtPAP2 is targeted to both chloroplasts and mitochondria. Its overexpression resulted in a higher miR173 abundance (Table 2) and tasiRNA biogenesis (Figure 1) from *TAS1* and *TAS2* loci (Figure 3), which may degrade their *PPR*/*TPR* target mRNAs, as reflected by the microarray data (Table 3). PPR proteins, characterized by tandemly arranged 35-amino acid repeats, are predominantly targeted to chloroplasts or mitochondria and take part in virtually all the processes in RNA processing and editing [38,39]. Further studies are required to understand how AtPAP2 overexpression affects PPR. Yeast-two-hybrid experiments showed that AtPAP2 can interact with a number of Multiple Organellar RNA editing factors (MORFs) (Yeesong LAW, unpublished data), which interact with PPR proteins to carry out RNA processing in chloroplasts and mitochondria [40]. Overexpression of *AtPAP2* may modulate certain RNA processing mechanisms in chloroplasts and mitochondria, thereby affecting the physiology of these two energy-generating organelles (Figure 4). RNA-seq of organeller RNA from the overexpression line will give us a better picture on this hypothesis.

Anthocyanin production can be induced in *Arabidopsis* by stresses such as phosphate deficiency [18] and sugar treatments [41]. Phosphate deficiency induces MYB transcription factors (MYB75/90/113), which subsequently activate the expression of multiple genes involved in anthocyanin synthesis. MYB75 also stimulates the expression of miR828, which initiates the production of TAS4-SiR81(-) from the *TAS4* locus. TAS4-SiR81(-) targets mRNAs of MYB transcription factors for cleavage and serves as a negative feedback loop of anthocyanin production [18]. In this study, the counts of miR828 are zero in all the four lines, whereas the basal abundance of TAS4-SiR81(-) is lower in the OE lines (1,728 and 3,698 reads) than the WT (13,783 reads). Microarray studies showed that the basal abundance of many genes of enzymes involved in anthocyanin synthesis decreased in the leaves of the OE lines, while the transcript abundance of MYB75/90/119 was indifferent [16]. These lower basal levels are physiologically relevant, as high sucrose (6%) treatment induced less anthocyanin production in the OE lines than in the WT [16]. Phosphate deficiency induced all the three MYB factors (MYB75/90/113) [18], but only exogenous sucrose treatment induced MYB75 [41]. In the lines, high endogenous sugars suppress the basal transcript abundance of enzymes, which are independent of the transcript abundance of the MYB transcription factors [16]. These data indicates that the induction of anthocyanin production by exogenous sucrose and phosphate deficiency are overlapped but distinguishable. In addition, since the count of miR828 is zero in all our samples, the cleavage of *TAS4* RNA maybe initiated by an uncharacterized sRNA, adding complexity to the regulatory pathway of anthocyanin production.

## Conclusion

Energy is the driving force of growth; various biological processes are regulated by the availability of energy. The AtPAP2 overexpression lines provide a unique opportunity to study the impact of high energy status on the regulatory mechanisms of plants. Our previous works showed that there are massive changes in the gene transcription profiles in the OE lines [16]. Small RNA (sRNA)-mediated cleavage of mRNA is the key mechanism to induce mRNA degradation. The present study is the first to report the sRNA expression profile of *Arabidopsis* when ample supply of energy is available. Significant changes in certain miRNAs and the miR173-tasiRNAs-*PPR*/*TPR* network were observed in the fast-growing lines. Further investigation is required to delineate the roles of the miR173-tasiRNAs network in plant growth and energy metabolism.

## Methods

### Plant materials

*Arabidopsis thaliana* ecotypes Columbia (Col-0) (wild type: WT), AtPAP2 mutant (*pap2*) in Col-0 background, AtPAP2 overexpressors (OE7 and OE21) in Col-0 background were used in this study [14]. *Arabidopsis* seeds of WT, *pap2* and OE lines were surface sterilized with 20% (v/v) bleach for 15 ~ 20 minutes and washed with sterilized water for 4 ~ 5 times. The seeds were plated on Murashige and Skoog medium supplemented with 2% (w/v) sucrose for 10 days and the seedling were transferred to soil under 16 h light (22°C)/8 h dark (18°C) period. 20-day-old *Arabidopsis* leaf and root without bolting were frozen immediately in liquid nitrogen for RNA extraction. For every plant line, individual leaf/root was mixed as one sample.

### Total RNA isolation

The total RNA of leaf and root were extracted with DNase I digestion according to the manufacturer’s instructions (RNeasy Plant Mini Kit, Qiagen, Hong Kong). The preliminarily quality was detected by running 1% (w/v) agarose gel stained by GelRed (Biotium, USA). The quality of the RNA samples was tested with Agilent 2100 Bioanalyzer RNA Nanochip. At least 10 μg of total RNA at a concentration of ≥ 400 ng/μl, OD 260/280 = 1.8 ~ 2.2 were used for cDNA library preparation.

### Small RNA sequencing and sequencing process

The total RNA was sent to Beijing Genomics Institute (BGI) (Shenzhen, Guangdong province, China), small RNAs were sequenced using Illumina HiSeq™ 2000 high throughput sequencing technology. sRNAs with the length of 18-30 nt were first separated from the total RNA by running on 15% (w/v) TBE urea polyacrylamide gel. The excised gel with sRNAs from 18 to 30 nt in length were submerged in 600 μL of 0.3 M sodium chloride overnight at 4°C. The sRNA fragments were dissolved in diethylpyrocarbonate treated water (Ambion, Austin, TX, USA). The sRNAs were then added 5′ adaptor by T4 RNA ligase (Takara, Dalian, China) in the presence of RNase OUT Ribonuclease Inhibitor (Invitrogen, China) overnight at 4°C according to the manual. After obtaining the 5′ ligation products, a 3′ adaptor was added to the selecting 5′ adaptor sRNAs following the same procedures as the 5′ adaptor ligation. Finally, both 3′ and 5′ adaptors ligated sRNAs were run on a 10% (w/v) TBE urea polyacrylamide gel, then the 44 nt RNAs were excised. The sRNA was reversely transcribed to cDNA with the RT primer by Superscript II reverse transcriptase (Invitrogen, China). The cDNA was further quantified by Agilent 2100 and sequenced by the Illumina 1G Genome Analyzer.

### Small RNAs annotations in NCBI and Rfam databases

Raw reads were produced by the Illumina 1G Genome Analyzer at BGI-Shenzhen, China and processed into clean full length reads by the BGI small RNA pipeline procedure [42]. During this procedure, all low quality reads including 3′ adapter reads and 5′ adapter contaminants were removed. Adapter sequences were trimmed off from the remaining high quality sequences and sequences lager than 30 nt and smaller than 18 nt were discarded. All high quality sequences were used for further analysis. Unique small RNA tags were first mapped to *Arabidopsis thaliana* genome (http://www.arabidopsis.org/, TAIR version 10) using SOAP software to analyze the expression and distribution on the genome [43]. Annotated small RNAs derived from rRNA, scRNA, snoRNA, snRNA and tRNA deposited at the Rfam 9.1 and NCBI GenBank databases (http://www.ncbi.nlm.nih.gov) were identified by NCBI blast. Besides, sRNA tags were also aligned to exons, introns, repeats and small interference RNAs. A final small RNA annotation was obtained by following a priority rule: Genbank > Rfam > known miRNA > repeat > exon > intron > siRNA. All the raw data and processed data were deposited in NCBI GEO (http://www.ncbi.nlm.nih.gov/geo/) with accession number GSE48309.

### Known miRNAs and novel miRNAs identification and targets prediction

In order to obtain known miRNAs abundance between different lines, unique sequences were aligned to the miRNA precursors in miRBase 15.0 (http://www.mirbase.org/) using tag2miRNA software developed by BGI. If the miRNAs had no precursors, mature miRNAs sequences were used for miRNAs alignment [44]. Then the sRNAs counts and miRNA IDs of every line were obtained and all the counts were normalized according to the normalization formula (Normalized expression = Actual miRNA count/Total count of clean reads*15,000,000). After comparing the known miRNAs abundance between two samples (treatment vs. control), log2-ratio scatter plot figures were plotted between the two compared samples with p-value < 0.05. In order to determine the significant differences of known sRNA between different lines, all reads were normalized and p-value was calculated by using the formulas listed as follows [45]. The first step for calculation was to use the formula:

$$p\left(\left(y\right|x\right)={\left(\frac{N_{2}}{N_{1}}\right)}^{y}\frac{\left(x+y\right)!}{x!y!{\left(1+\frac{N_{2}}{N_{1}}\right)}^{\left(x+y+1\right)}}$$

Where N1 is the total clean reads without normalization in control sample, N2 is the total reads in treatment, x is the number of a certain sRNA in control library and y is the number of the same sRNA in over-expressed library. A two-tailed p-value was calculated as p = 2q, where q is the accumulated probability $q=\sum_{y^{'}=0}^{y^{'}\leq y}p\left(\left(y^{'}\right|x\right)$. If q is larger than 0.5, p = 2*(1-q).

MIREAP (http://sourceforge.net/projects/mireap/) was used to identify potential novel miRNAs by folding the flaking genome sequence of unique small RNAs. Target genes are predicted based on the rules suggested by Allen et al [5] and Schwab [21]. All the novel miRNA candidates and their targets were identified according to the following criteria: (1) there should be no more than four mismatches between the sRNAs and their targets, (2) in positions 2-12 of the miRNA/target duplex, there should be no adjacent mismatches, (3) no more than two adjacent mismatches in the miRNA/target duplex, (4) in positions 10-11 of miRNA/target duplex, there should be no mismatches, (5) in 5′ end of miRNAs, the mismatches in position 1-12 should no more than 2.5 basepairs with their targets (G-U bases count as 0.5 mismatches), and (6) the Minimum free energy (MFE) value of miRNA/target duplex should be no less than 75% of the value of the same miRNA matched to its perfect target.

### Tasi-RNAs analysis

All the 21-nt sRNA clean reads (5′-3′direction) from each library were mapped to the eight *TAS* cDNA sequences (5′-3′direction) using SOAP3 software [46] with perfect match. The reads were normalized and the fold change (log2-ratio) and the p-value were calculated between OE and WT lines. For phasing register analysis, we plotted 21nt-phasing register for each *TAS* cDNA based on the count and position information. The network in Figure 3 was generated by using Cytoscape software. miR173, all 21-nt small RNAs that are perfected mapped to *TAS* loci, all potential *PPR*, *TPR* targets, *TAS1A*, *1B*, *1C*, *TAS2* and *MIR173* genes are imported as nodes. The 21-nt small RNAs and their potential targets were connected with edges, as well as the mapped *TAS* genes. miR173 was connected to *MIR173* gene and *TAS1A*, *1B*, *1C* and *TAS2*, respectively. The network is clustered by using the Cytoscape cluster plugin. The sRNA data were fit into the miR173-tasiRNAs-*PPR* network using Cytoscape 3.0 [47].

### NatsiRNAs analysis

Databases of cis- and tran-NATs pairs (TAIR version 10, http://www.arabidopsis.org) were provided by Prof. Weixiong Zhang (Washington University in Saint Louis). The mapping procedure was listed in Additional file 15. Based on gene-pair names and genomic locations, we were able to align clean reads to *Arabidopsis* genome and crosscheck the database to find the natsiRNAs with significant differences in abundance between the lines. For each gene-pair, the start and the end for overlapping region (OL) and the whole region (WL) of two OL genes were used for mapping by SOAP3 software. Both strands were used in the mapping. If the sequence of a read is identical to part of either strand with the same length from 5′ to 3′ direction, then the read is perfectly matched. If the read is transcribed from positive strand, then its sequence is identical to the negative strand; therefore, its mapping orientation is denoted as negative (-), otherwise denoted as positive (+). For cis-NATs mapping, all clean reads from the 8 libraries from both leaf and root were mapped to *Arabidopsis* cis-NATs pairs regions with no mismatch allowed and all mapping positions were kept for each read. Finally we counted the number of reads aligned within each OL and WL region for all lines, and computed fold change and p-value between WT and OE lines.

### Quantitative RT-PCR

Quantitative reverse transcriptase PCR (qRT-PCR) analysis was carried out using cDNA samples transcribed from 20-day-old tissues of *Arabidopsis*. Primer premier 5.0 (http://www.premierbiosoft.com/primerdesign/) was used to design the qRT-PCR primers. The PCR reactions were performed in a 10 μL volume containing a 2 × SYBR Green Master Mix (ABI systems). The amplification parameters were 95°C for 1 min; followed by 40 cycles of 95°C, 15 s and 60°C 1 min. ACTIN 2 was used as the internal control. For every transcript, each cDNA sample was analyzed in triplicate, and relative transcript abundance was calculated by normalizing to the maximum amount of concentration. The assessment of expression comparing different targets was determined by the ddCt comparative threshold (ΔΔCt) method. p-values were determined by a two-tailed paired Student’s *t* test. sRNAs were transcribed using the miRNA First-Strand Synthesis Kit (Clontech) and qRT-PCR of sRNA was carried out at the same condition of the qRT-PCR of mRNA. All the primers used in this study were listed in Additional file 16.

## Competing interests

The authors declare that they have no competing interests.

## Authors’ contributions

CL designed the experiments, sample collection, data analysis, and drafted the manuscript. XL participated in the analysis of tasiRNA and natsiRNA using computational methods and generated the miR173-tasiRNAs-*PPR*/*TPR* network. YS carried out qRT-PCR experiments. SY participated in the data analysis and provided helpful suggestions, and BLL was responsible for the overall concept and revising manuscript. All authors read and approved the manuscript.

## Supplementary Material

Additional file 1 Statistics of small RNA sequences from eight libraries. Click here for file

Additional file 2 Size distribution of small RNA sequences in various leaf (a) and root (b) samples. Click here for file

Additional file 3 Classification of sRNAs in various samples. Click here for file

Additional file 4 **Full list of all known miRNAs in *Arabidopsis***. Click here for file

Additional file 5 Structures of novel miRNAs in leaf and root. Click here for file

Additional file 6 List of novel miRNAs and their predicted targets. Click here for file

Additional file 7 **List of novel *MIR *** **genes discovered in this study.** Click here for file

Additional file 8 List of tasiRNAs that are significantly altered in both OE lines. Click here for file

Additional file 9 Phase register of tasiRNAs. Click here for file

Additional file 10 ***PPR*/*TPR *** **genes in miR173-tasiRNAs-*PPR/TPR *** **network.** Click here for file

Additional file 11 Full list of expression of cis-nat-siRNAs that mapped to the cis-nat-siRNA pairs from TAIR version10. Click here for file

Additional file 12 Full list of expression of trans-nat-siRNAs that mapped to the trans-nat-siRNA pairs from TAIR version10. Click here for file

Additional file 13 Validation of small RNAs and microarray data by qRT-PCR. Click here for file

Additional file 14 Microarray data of all PPR proteins encoding genes. Click here for file

Additional file 15 **Flowchart of small RNAs mapped to cis-NATs and trans-NATs in *Arabidopsis *** **by SOAP3.** Click here for file

Additional file 16 Primers used in Quantitative RT-PCR validation. Click here for file
