# Supplementary material for: Global small RNA analysis in fast-growing Arabidopsis thaliana with elevated concentrations of ATP and sugars
Source: BMC Genomics. 2014 Feb 10;15:116. doi: 10.1186/1471-2164-15-116 (PMC3925372; doi:10.1186/1471-2164-15-116)
Supplement: Additional file 1 — Statistics of small RNA sequences from eight libraries. [file 1471-2164-15-116-S1.pdf]

Additional file 1. Statistics of small RNA sequences from eight libraries.

Library	Raw reads (R)	Clean reads (C)		Mapped to genome (M)	
	Total	Total (C/R%)	Unique (C/R%)	Total (M/C%)l	Unique (M/C%)
**Leaf**					
WT	16,462,993	15,631,599 (94.95%)	1,987,787 (12.07%)	13,640,806 (87.26%)	1,322,443 (66.53%)
pap2	14,699,954	14,121,529 (96.07%)	2,011,308 (13.68%)	12,265,644 (86.86%)	1,342,451 (66.75%)
OE7	18,345,272	17,331,077 (94.47%)	2,024,161 (11.03%)	13,388,975 (77.25%)	922,738 (45.59%)
OE21	16,738,094	16,169,858 (96.61%)	2,176,249 (13.00%)	12,597,144 (77.91%)	1,015,795 (46.68%)
**Root**					
WT	13,154,174	12,500,900 (95.03%)	1,690,713 (12.85%)	9,678,185 (77.42%)	564,994 (33.42%)
pap2	16,444,289	15,579,209 (94.74%)	2,114,466 (12.86%)	11,472,399 (73.64%)	618,737 (29.26%)
OE7	15,990,223	15,314,996 (95.78%)	1,590,360 (9.95%)	12,435,120 (81.20%)	483,788 (30.42%)
OE21	17,410,769	16,642,942 (95.59%)	2,242,479 (12.88%)	12,129,420 (72.88%)	499,766 (22.29%)
