# Supplementary figures and images for: Global small RNA analysis in fast-growing Arabidopsis thaliana with elevated concentrations of ATP and sugars
Source: BMC Genomics. 2014 Feb 10;15:116. doi: 10.1186/1471-2164-15-116 (PMC3925372; doi:10.1186/1471-2164-15-116)

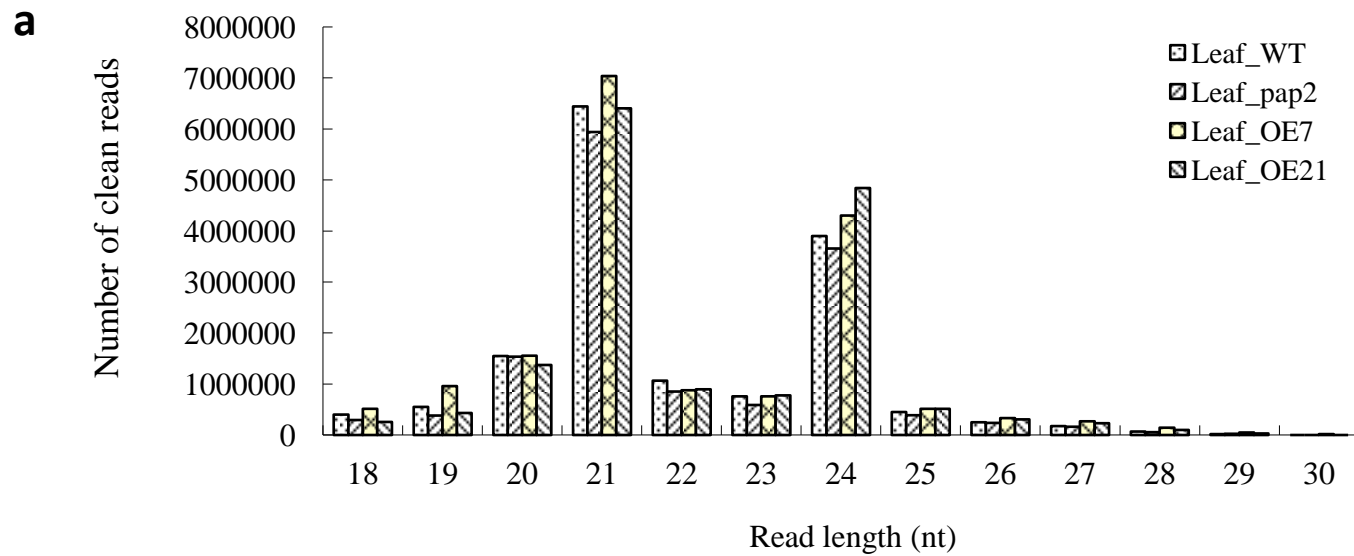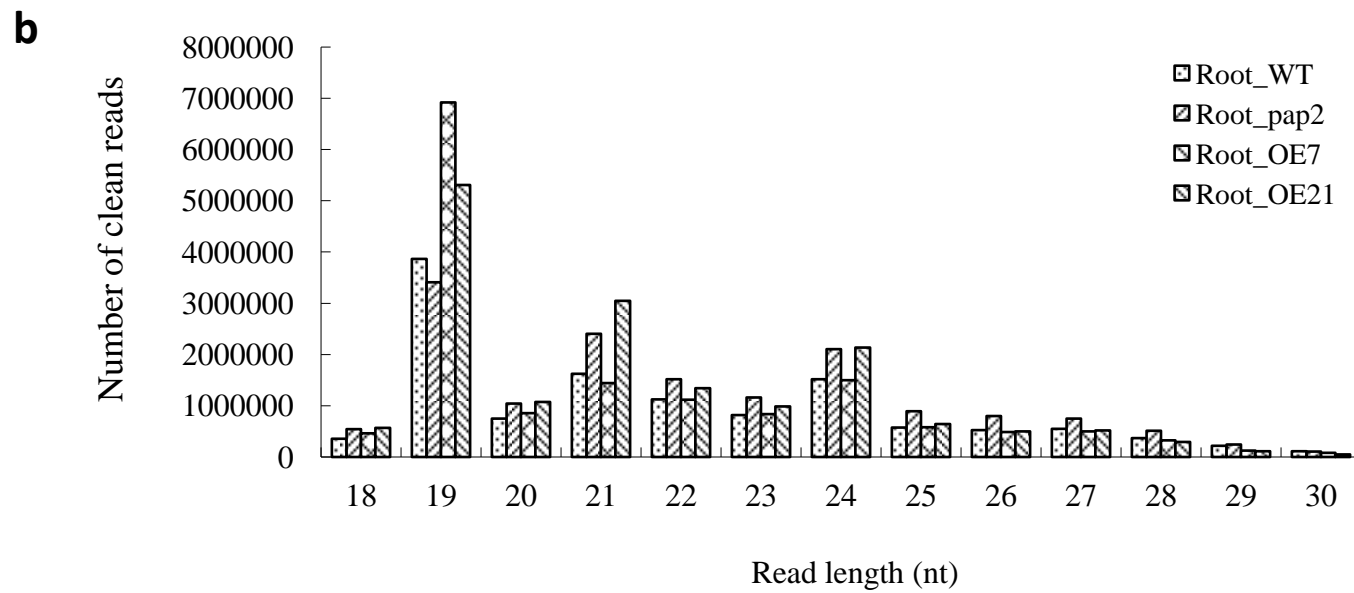

**Additional file 2:** Size distribution of small RNA sequences in various leaf (a) and root (b) samples.

Supplement: Additional file 2 — Size distribution of small RNA sequences in various leaf (a) and root (b) samples. [file 1471-2164-15-116-S2.pdf]
