# Supplementary material for: Global small RNA analysis in fast-growing Arabidopsis thaliana with elevated concentrations of ATP and sugars
Source: BMC Genomics. 2014 Feb 10;15:116. doi: 10.1186/1471-2164-15-116 (PMC3925372; doi:10.1186/1471-2164-15-116)
Supplement: Additional file 3 — Classification of sRNAs in various samples. [file 1471-2164-15-116-S3.pdf]

**Additional file 3. Classification of sRNAs in various samples.**

| **Total reads** Category | Leaf WT% | Leaf *pap2*% | Leaf OE7% | Leaf OE21% | Root WT% | Root *pap2*% | Root OE7% | Root OE21% |
|---|---|---|---|---|---|---|---|---|
| exon_antisense | 0.87 | 0.99 | **1.63** | **1.84** | 0.25 | 0.23 | 0.29 | **0.43** |
| exon_sense | 2.33 | 2.39 | **1.98** | 2.29 | 1.76 | 1.62 | 1.9 | 1.57 |
| intron_antisense | 0.2 | 0.23 | 0.2 | 0.26 | 0.06 | 0.06 | **0.03** | 0.07 |
| intron_sense | 0.27 | 0.28 | 0.16 | 0.2 | 0.09 | 0.09 | **0.05** | 0.08 |
| miRNA | 37.5 | 41.02 | 35.24 | 30.77 | 5.95 | 7.03 | **3.45** | 6.04 |
| rRNA | 21.32 | 15.65 | 19.91 | 18.8 | 36.47 | 42.47 | 28.94 | 28.25 |
| repeat | 12.39 | 12.85 | **7.77** | 8.67 | 3.87 | 4.15 | **1.97** | 2.86 |
| siRNA | 1.78 | 2.68 | **3.96** | **8.99** | 0.39 | 0.54 | 0.57 | **6.08** |
| snRNA | 0.06 | 0.05 | 0.07 | 0.06 | 0.19 | 0.22 | 0.22 | 0.17 |
| snoRNA | 0.03 | 0.03 | 0.04 | 0.04 | 0.03 | 0.04 | **0.05** | 0.04 |
| tRNA | 4.47 | 3.6 | **6.96** | 3.86 | 32.04 | 21.28 | 47.3 | 32.19 |
| unann | 18.77 | 20.23 | 22.07 | 24.23 | 18.92 | 22.27 | 15.24 | 32.19 |
| Total | 100 | 100 | 100 | 100 | 100 | 100 | 100 | 100 |

| **Unique reads** Category | Leaf WT% | Leaf *pap2*% | Leaf OE7% | Leaf OE21% | Root WT% | Root *pap2*% | Root OE7% | Root OE21% |
|---|---|---|---|---|---|---|---|---|
| exon_antisense | 2.22 | 2.16 | 1.7 | 1.67 | 0.89 | 0.82 | 0.65 | 0.63 |
| exon_sense | 7.86 | 7.73 | 6.4 | 6.96 | 9.4 | 8.02 | 14.02 | 7.67 |
| intron_antisense | 0.51 | 0.55 | 0.36 | 0.38 | 0.19 | 0.18 | 0.12 | 0.13 |
| intron_sense | 0.56 | 0.57 | 0.42 | 0.43 | 0.28 | 0.24 | 0.23 | 0.18 |
| miRNA | 0.16 | 0.15 | 0.14 | 0.13 | 0.1 | 0.08 | 0.1 | 0.08 |
| rRNA | 6.91 | 6.19 | 7.85 | 6.57 | 13.45 | 12.94 | 12.49 | 11.12 |
| repeat | 30.19 | 30.39 | 19.25 | 19.29 | 11.1 | 9.69 | 6.63 | 6.52 |
| siRNA | 1.54 | 1.53 | 2.07 | 2.33 | 0.5 | 0.56 | 0.61 | 0.8 |
| snRNA | 0.15 | 0.13 | 0.17 | 0.13 | 0.31 | 0.3 | 0.4 | 0.25 |
| snoRNA | 0.1 | 0.09 | 0.12 | 0.11 | 0.1 | 0.1 | 0.15 | 0.11 |
| tRNA | 1.08 | 1.05 | 1.28 | 0.96 | 2.75 | 2.68 | 2.7 | 2.61 |
| unann | 48.73 | 49.46 | 60.23 | 61.03 | 60.92 | 64.38 | 61.9 | 69.9 |
| Total | 100 | 100 | 100 | 100 | 100 | 100 | 100 | 100 |

Numbers in bold mean significant changes in OE versus WT, p-value < 0.05 and fold change log2 (OE/WT) ≥1 or ≤ -1.
