# Supplementary material for: Global small RNA analysis in fast-growing Arabidopsis thaliana with elevated concentrations of ATP and sugars
Source: BMC Genomics. 2014 Feb 10;15:116. doi: 10.1186/1471-2164-15-116 (PMC3925372; doi:10.1186/1471-2164-15-116)
Supplement: Additional file 5 — Structures of novel miRNAs in leaf and root. [file 1471-2164-15-116-S5.pdf]

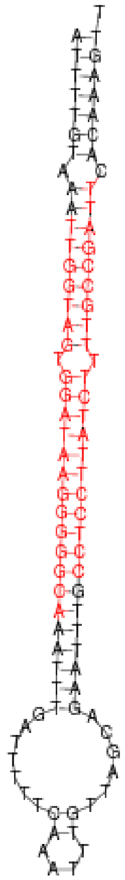

Leaf_miRNA0001
Root_miRNA0001

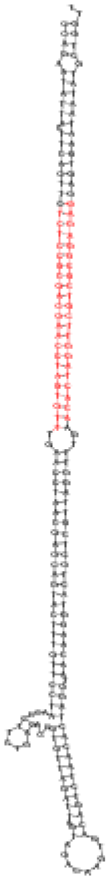

Leaf_miRNA0002
Root_miRNA0002

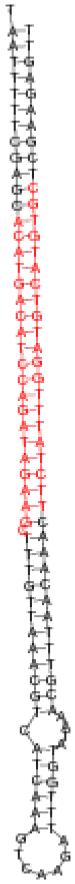

Leaf_miRNA0003

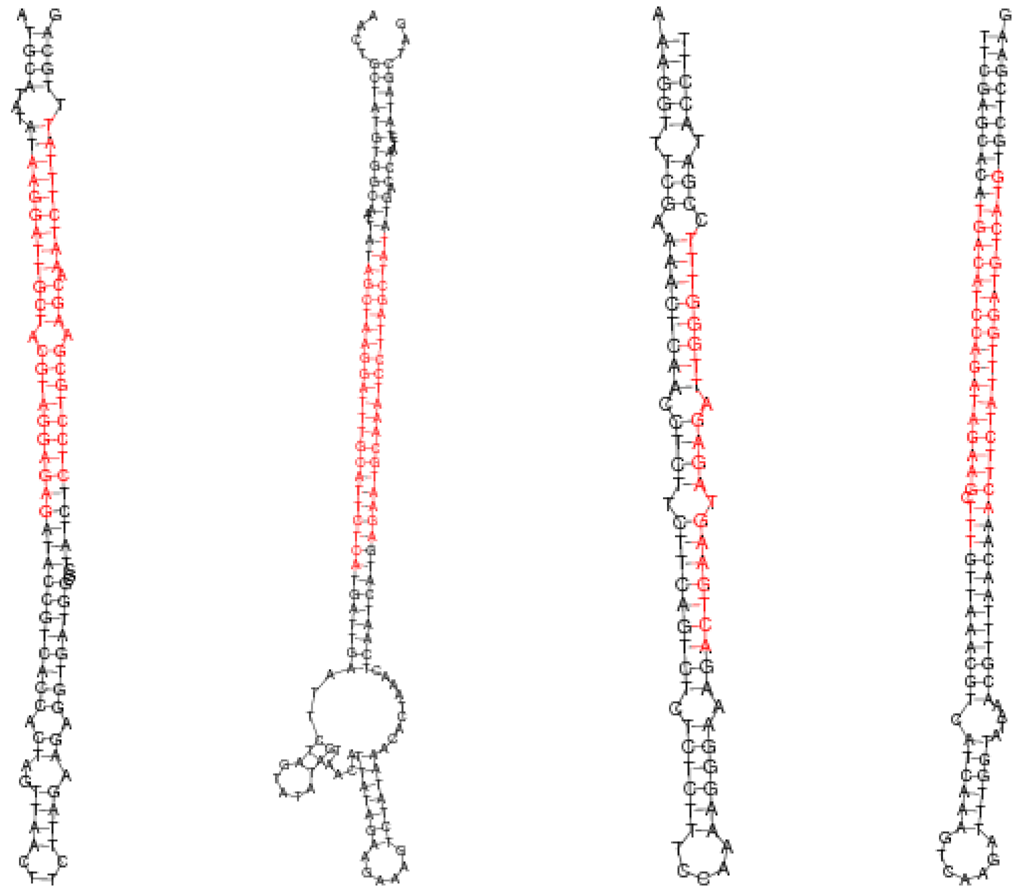

Leaf_miRNA0004 Leaf_miRNA0005 Leaf_miRNA0006 Leaf_miRNA0007

Additional file 5. Structures of novel miRNAs in leaf and root. The mature miRNAs were shown in red letters.
