# Supplementary material for: Global small RNA analysis in fast-growing Arabidopsis thaliana with elevated concentrations of ATP and sugars
Source: BMC Genomics. 2014 Feb 10;15:116. doi: 10.1186/1471-2164-15-116 (PMC3925372; doi:10.1186/1471-2164-15-116)
Supplement: Additional file 6 — List of novel miRNAs and their predicted targets. [file 1471-2164-15-116-S6.pdf]

Additional file 6. List of novel miRNAs and their predicted targets

Name	WT	*pap2*	OE7	OE21	Predicted target genes	Gene family
Leaf_miRNA0001_5p[a]	583	501	409	396	AT1G80740	Chromomethylase 1(CMT1)
Root_miRNA0001_5p[a]	403	645	505	706	AT1G80740	Chromomethylase 1(CMT1)
Leaf_miRNA0002_5p[b]	422	0	**0**	**0**	AT3G30820	Retrotransposon ORF-1 protein
Root_miRNA0002_3p[b]	108	90	**0**	**0**	AT3G30820	Retrotransposon ORF-1 protein
Leaf_miRNA0003_3p	0	3485	0	0	AT3G59230	F-box family protein
					AT5G27750	F-box family protein
Leaf_miRNA0004_5p	122	170	142	83	AT1G11580	Methylesterase PCR A (PMEPCRA)
Leaf_miRNA0005_5p	47	81	**95**	**139**	AT4G28010	Pentatricopeptide (PPR) repeat-containing protein
Leaf_miRNA0006_3p	0	0	**364**	**315**	AT1G62260	Pentatricopeptide (PPR) repeat-containing protein
Leaf_miRNA0007_5p	1920	0	**0**	**0**	AT1G58310	F-box family protein
					AT3G59230	F-box family protein
					AT4G00320	F-box family protein
					AT5G41840	F-box family protein

[a,b] Novel miRNAs from Leaf and root represented by the same letter are identical. Numbers in bold mean significant changes in OE versus WT, p-value < 0.05 and fold change log2 (OE/WT) $\geq$1 or fold change log2 (OE/WT) $\leq$-1. The reads cut off is 100 and all reads were normalized.
