# Supplementary material for: Global small RNA analysis in fast-growing Arabidopsis thaliana with elevated concentrations of ATP and sugars
Source: BMC Genomics. 2014 Feb 10;15:116. doi: 10.1186/1471-2164-15-116 (PMC3925372; doi:10.1186/1471-2164-15-116)
Supplement: Additional file 9 — Phase register of tasiRNAs. [file 1471-2164-15-116-S9.pptx]

## Slide 1
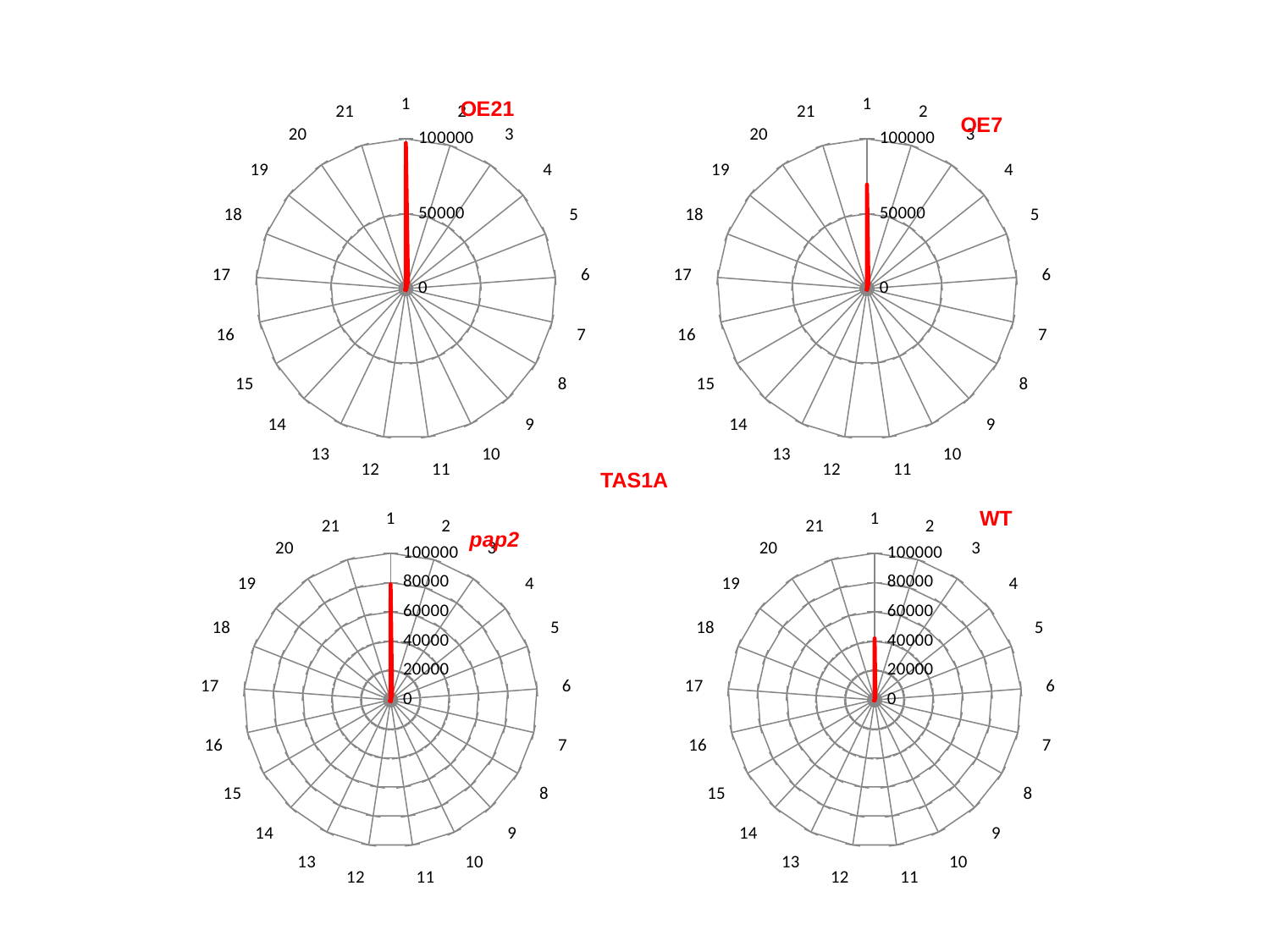

### Chart: OE21
| Category | S21_Sum |
|---|---|
### Chart: OE7
| Category | S7_Sum |
|---|---|
### Chart: pap2
| Category | ST_Sum |
|---|---|
### Chart: WT
| Category | SW_Sum |
|---|---|TAS1A

## Slide 2
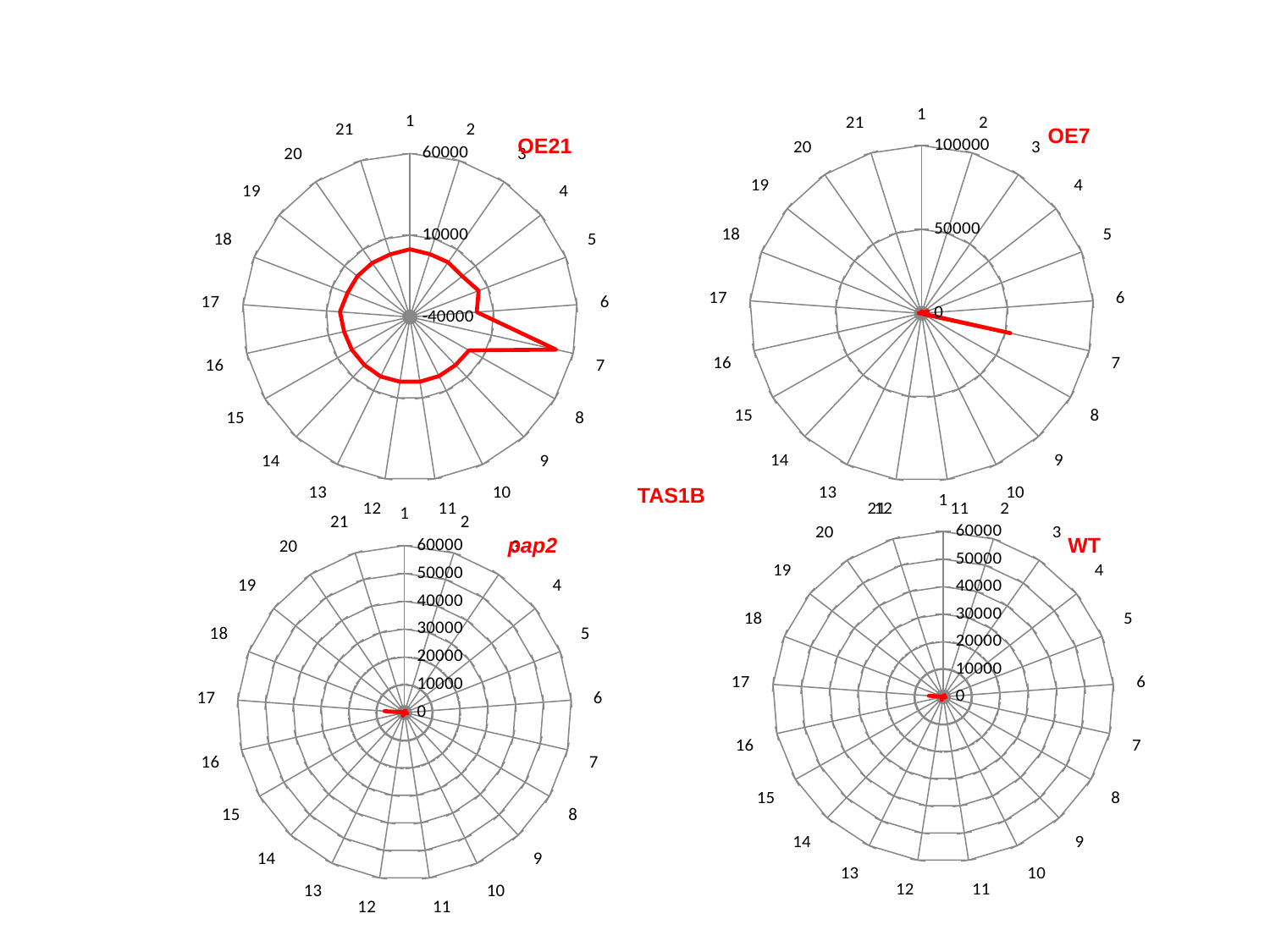

### Chart
| Category | S7_Sum |
|---|---|
### Chart
| Category | S21_Sum |
|---|---|OE7
OE21
### Chart
| Category | ST_Sum |
|---|---|TAS1B
### Chart
| Category | SW_Sum |
|---|---|pap2
WT

## Slide 3
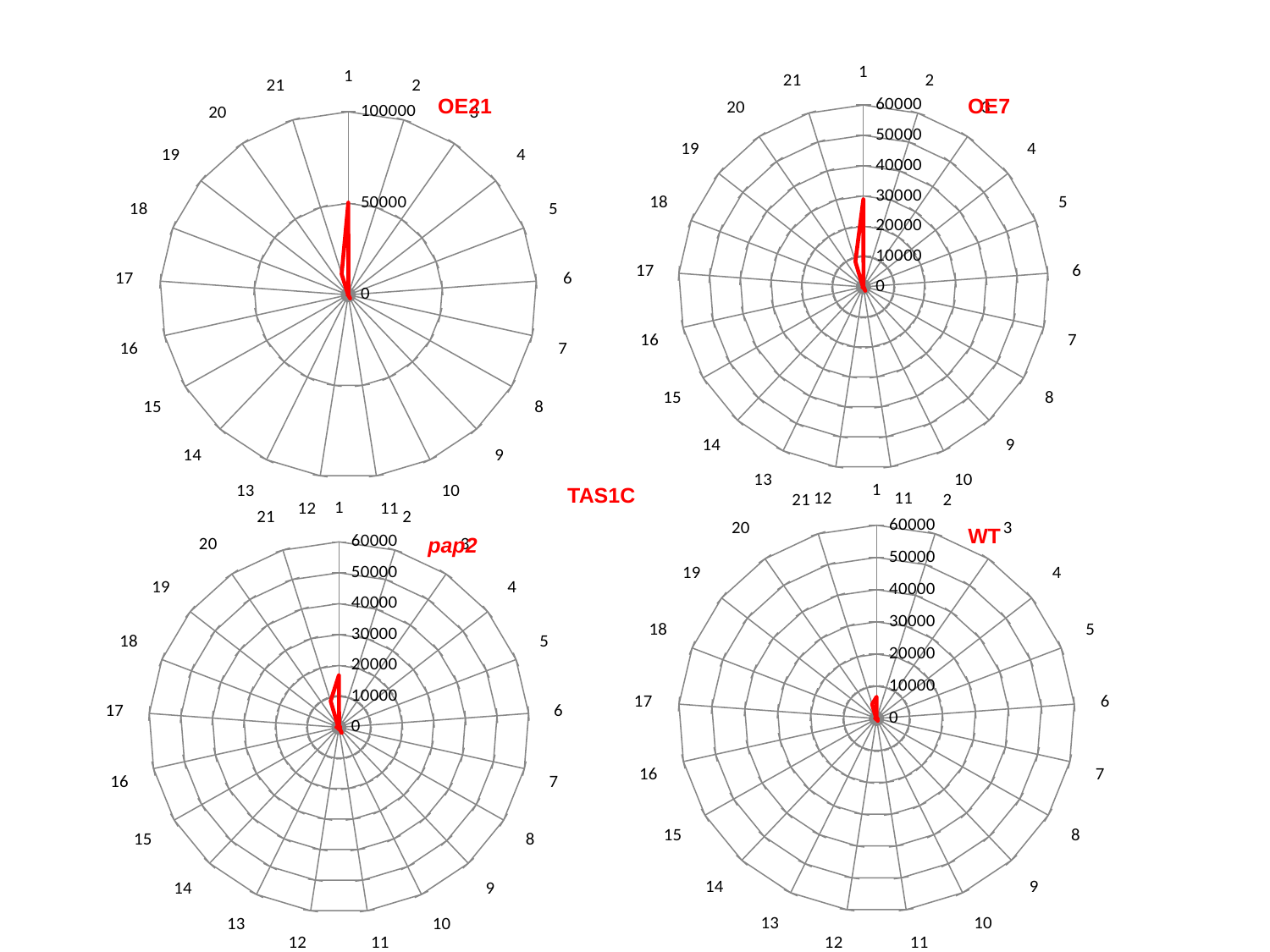

### Chart
| Category | S21_Sum |
|---|---|
### Chart
| Category | S7_Sum |
|---|---|OE21
OE7
### Chart
| Category | SW_Sum |
|---|---|TAS1C
### Chart
| Category | ST_Sum |
|---|---|WT
pap2

## Slide 4
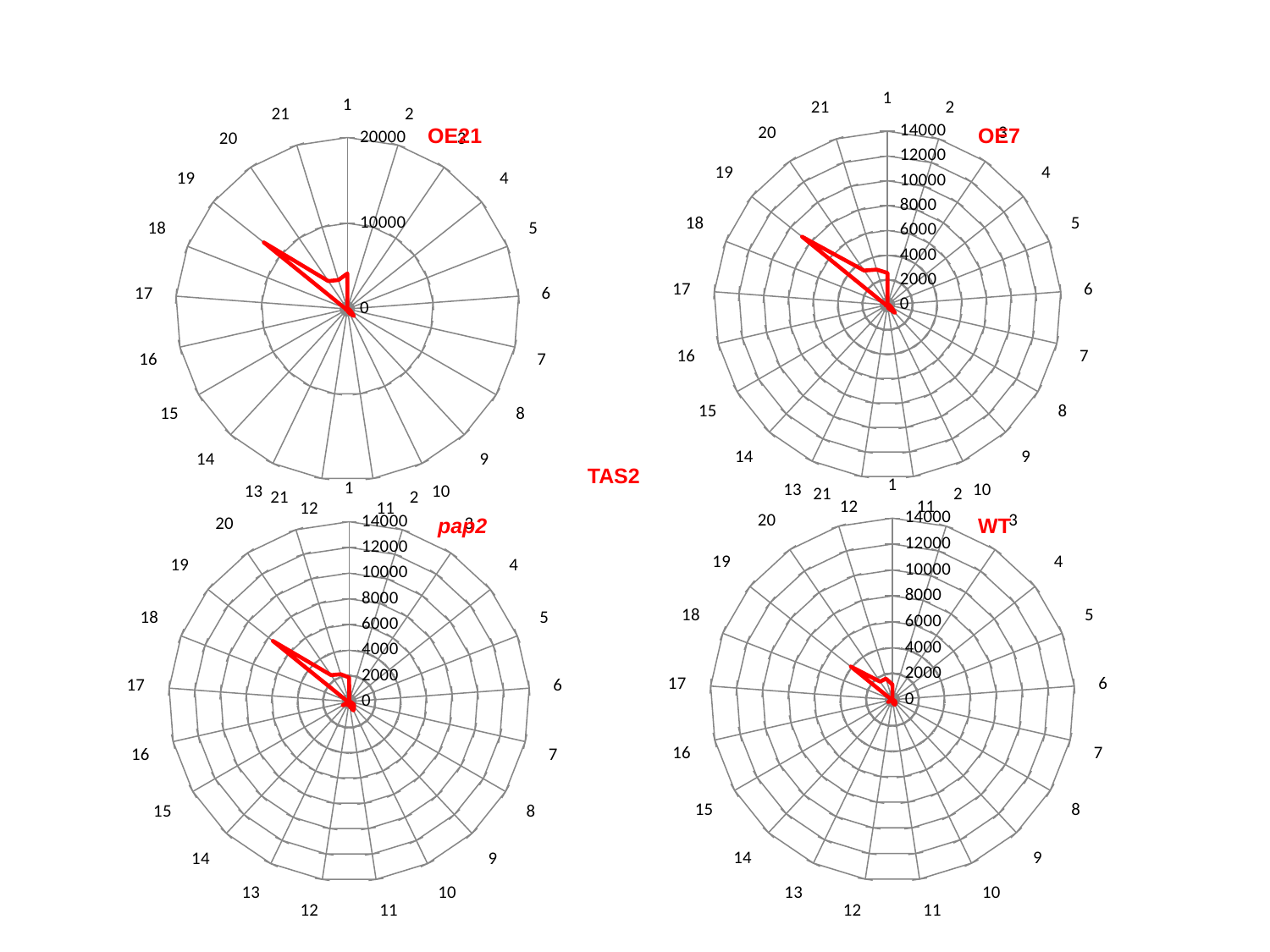

### Chart
| Category | S7_Sum |
|---|---|
### Chart
| Category | S21_Sum |
|---|---|OE21
OE7
### Chart
| Category | SW_Sum |
|---|---|TAS2
### Chart
| Category | ST_Sum |
|---|---|pap2
WT

## Slide 5
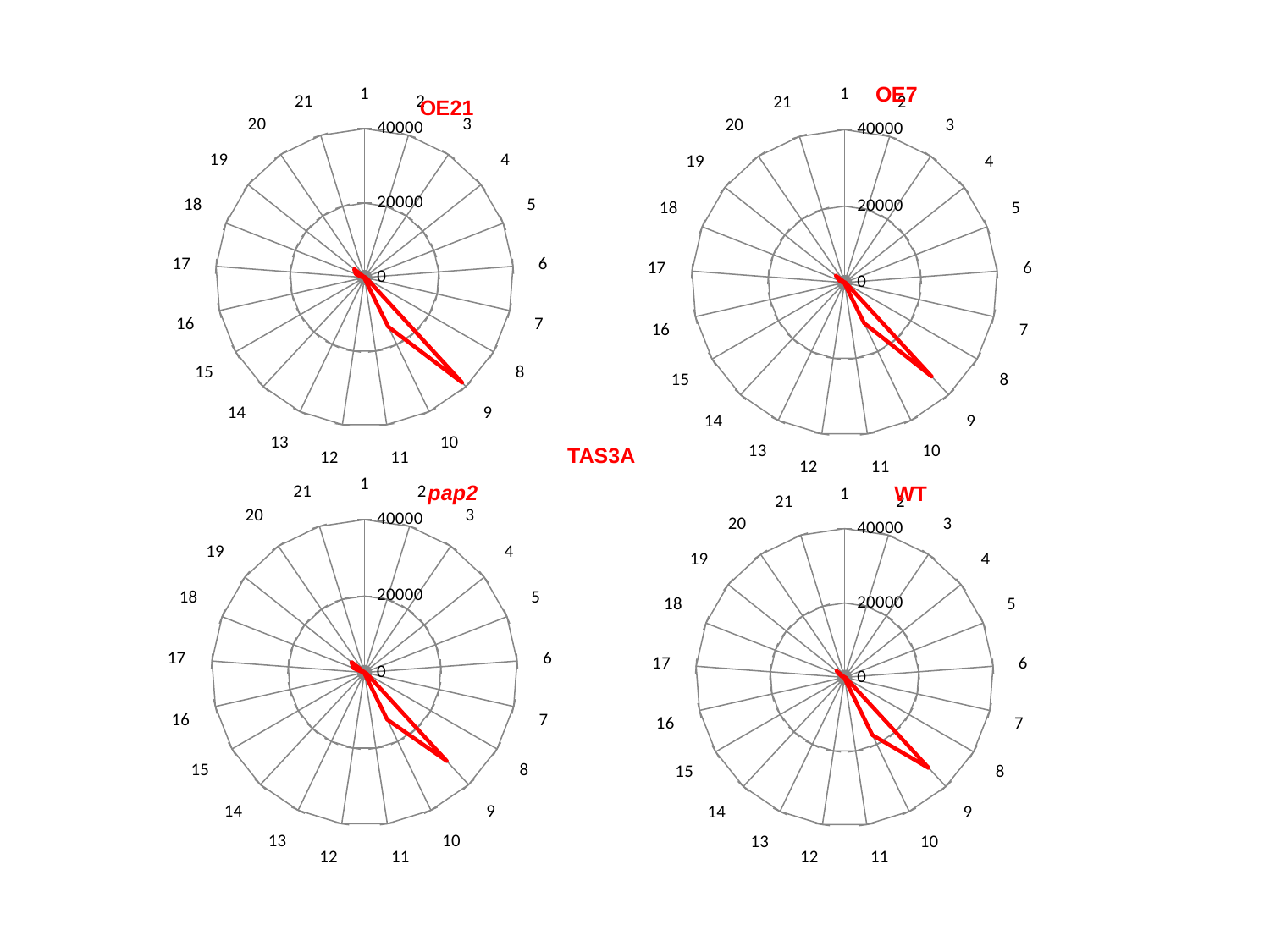

### Chart: OE21
| Category | S21_Sum |
|---|---|
### Chart: OE7
| Category | S7_Sum |
|---|---|
### Chart: pap2
| Category | ST_Sum |
|---|---|
### Chart: WT
| Category | SW_Sum |
|---|---|TAS3A

## Slide 6
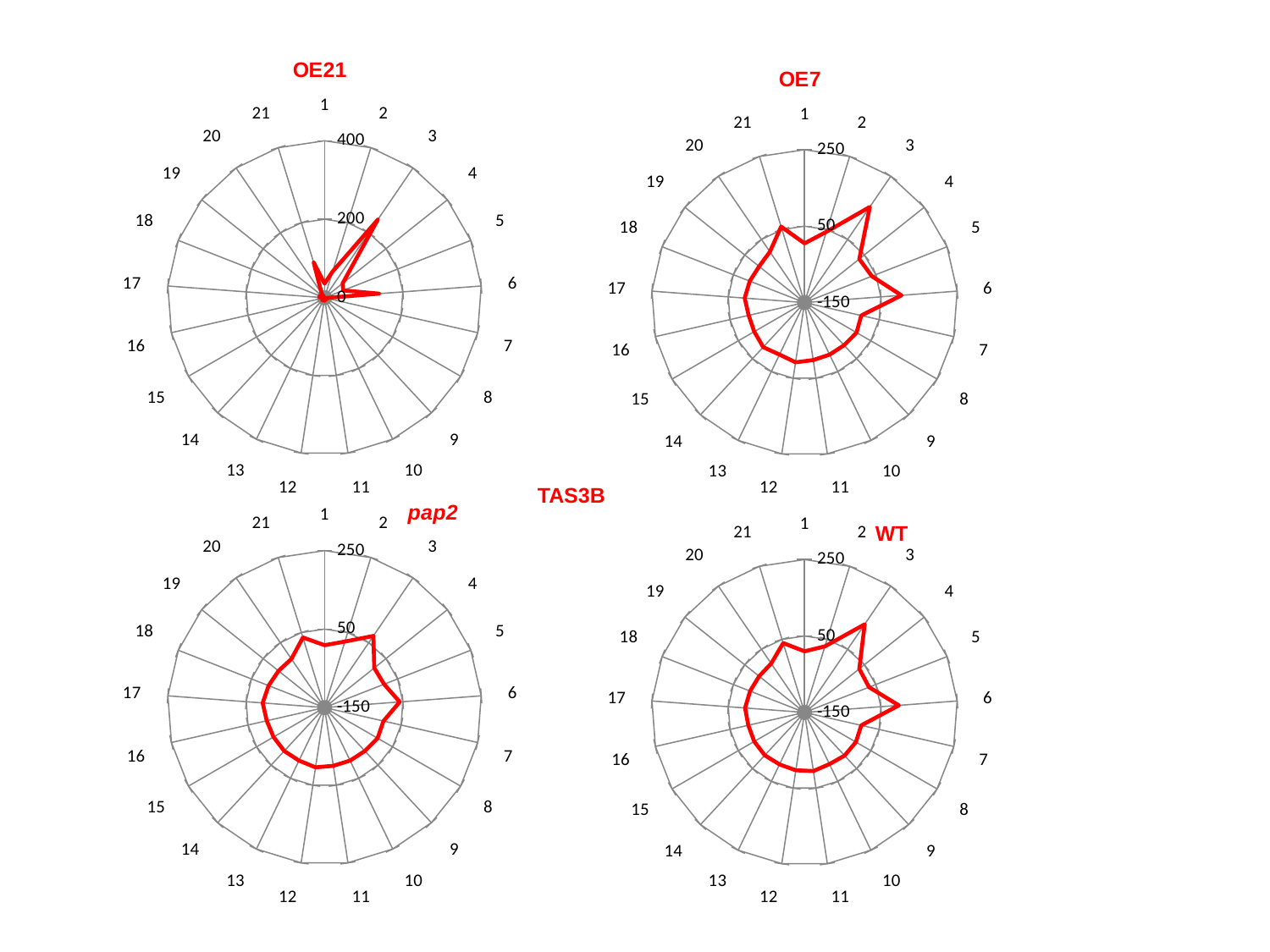

### Chart: OE21
| Category | S21_Sum |
|---|---|
### Chart: OE7
| Category | S7_Sum |
|---|---|
### Chart: pap2
| Category | ST_Sum |
|---|---|
### Chart: WT
| Category | SW_Sum |
|---|---|TAS3B

## Slide 7
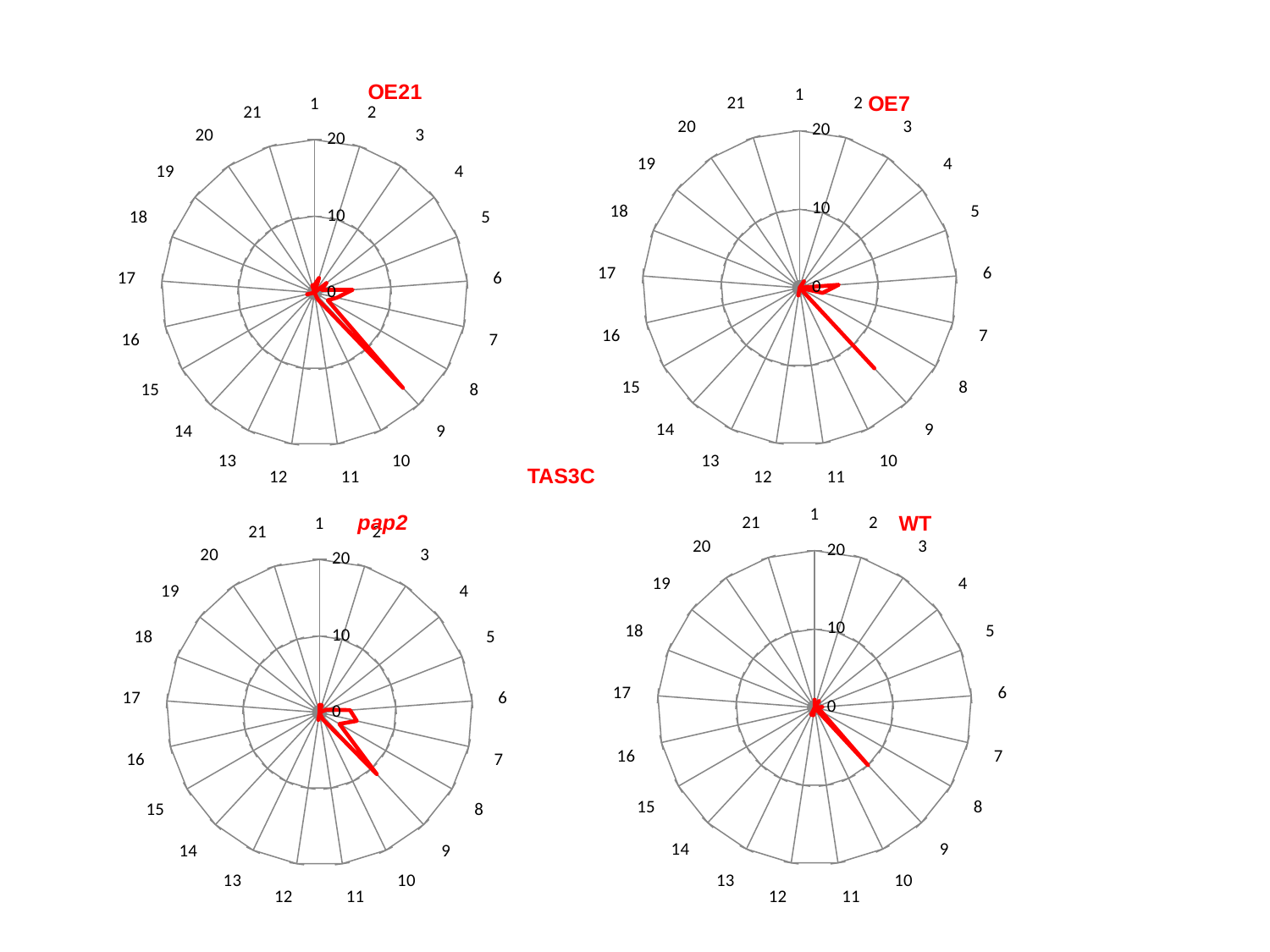

### Chart: OE7
| Category | S7_Sum |
|---|---|
### Chart: OE21
| Category | S21_Sum |
|---|---|
### Chart: WT
| Category | SW_Sum |
|---|---|
### Chart: pap2
| Category | ST_Sum |
|---|---|TAS3C

## Slide 8
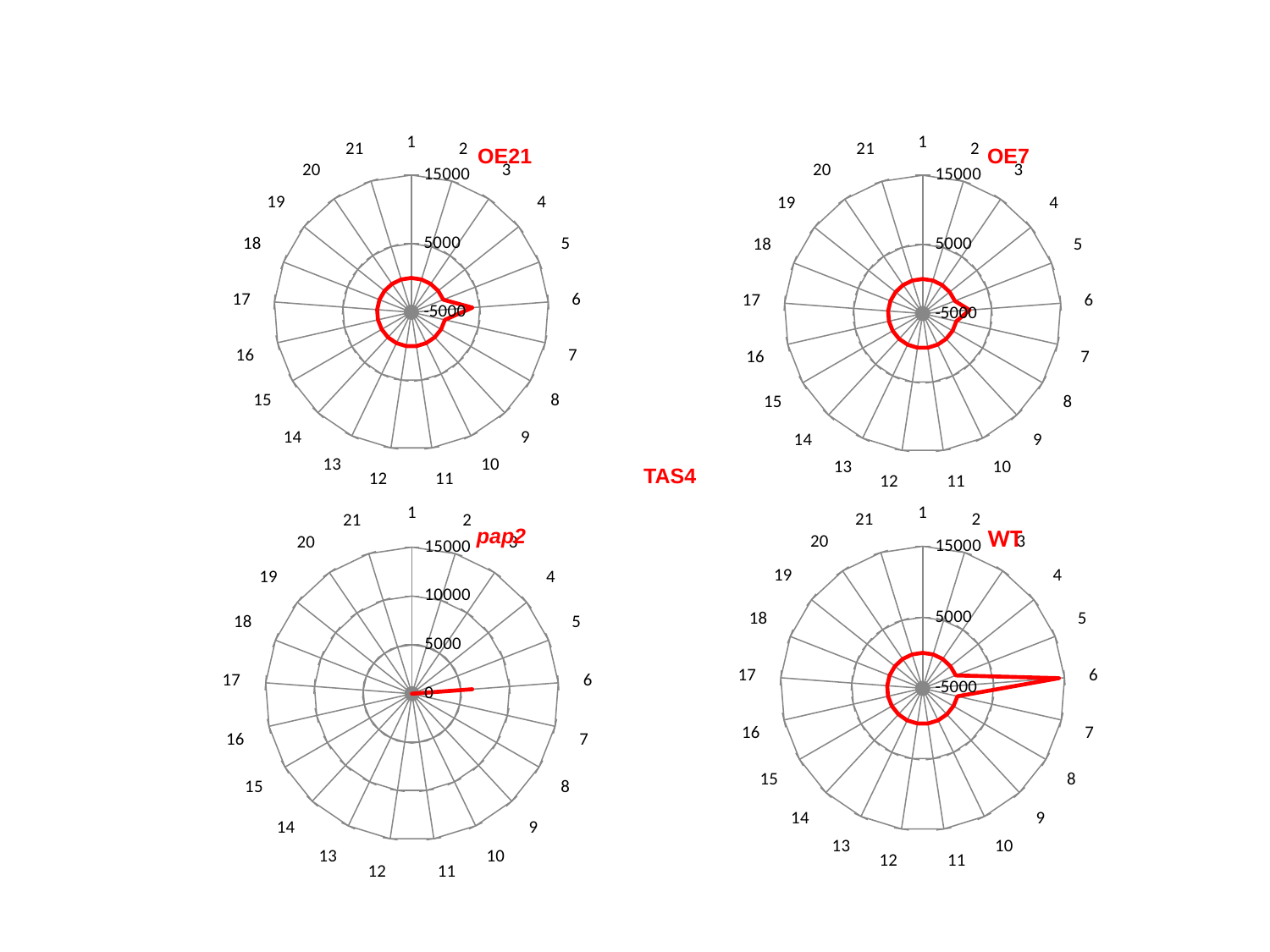

### Chart
| Category | S21_Sum |
|---|---|
### Chart
| Category | S7_Sum |
|---|---|OE21
OE7
TAS4
### Chart
| Category | ST_Sum |
|---|---|
### Chart
| Category | SW_Sum |
|---|---|pap2
WT
