# Supplementary material for: Global small RNA analysis in fast-growing Arabidopsis thaliana with elevated concentrations of ATP and sugars
Source: BMC Genomics. 2014 Feb 10;15:116. doi: 10.1186/1471-2164-15-116 (PMC3925372; doi:10.1186/1471-2164-15-116)
Supplement: Additional file 13 — Validation of small RNAs and microarray data by qRT-PCR. [file 1471-2164-15-116-S13.pdf]

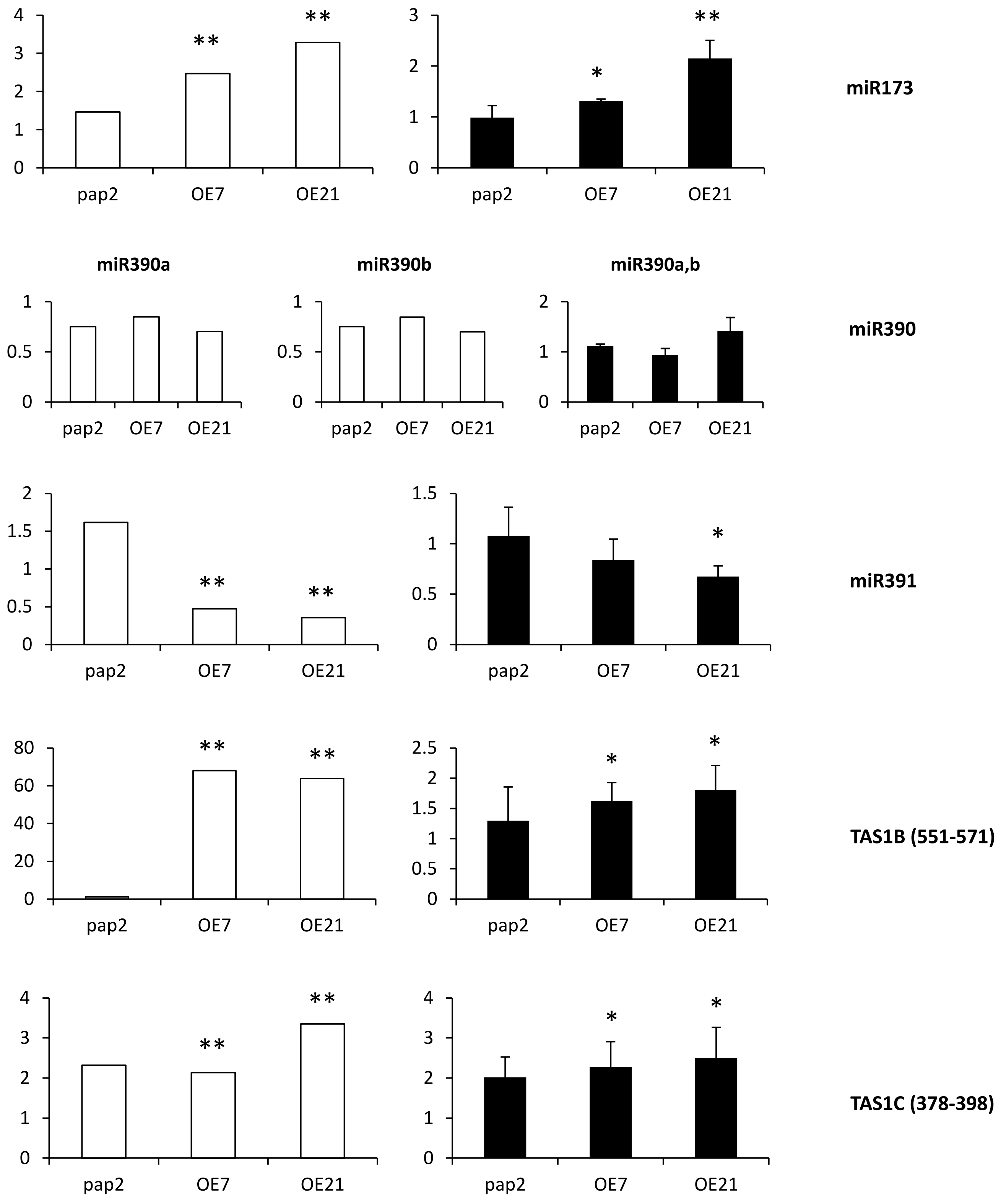
miR173
miR390a
miR390b
miR390a,b
miR390
miR391
TAS1B (551-571)
TAS1C (378-398)
pap2
OE7
OE21
**
*

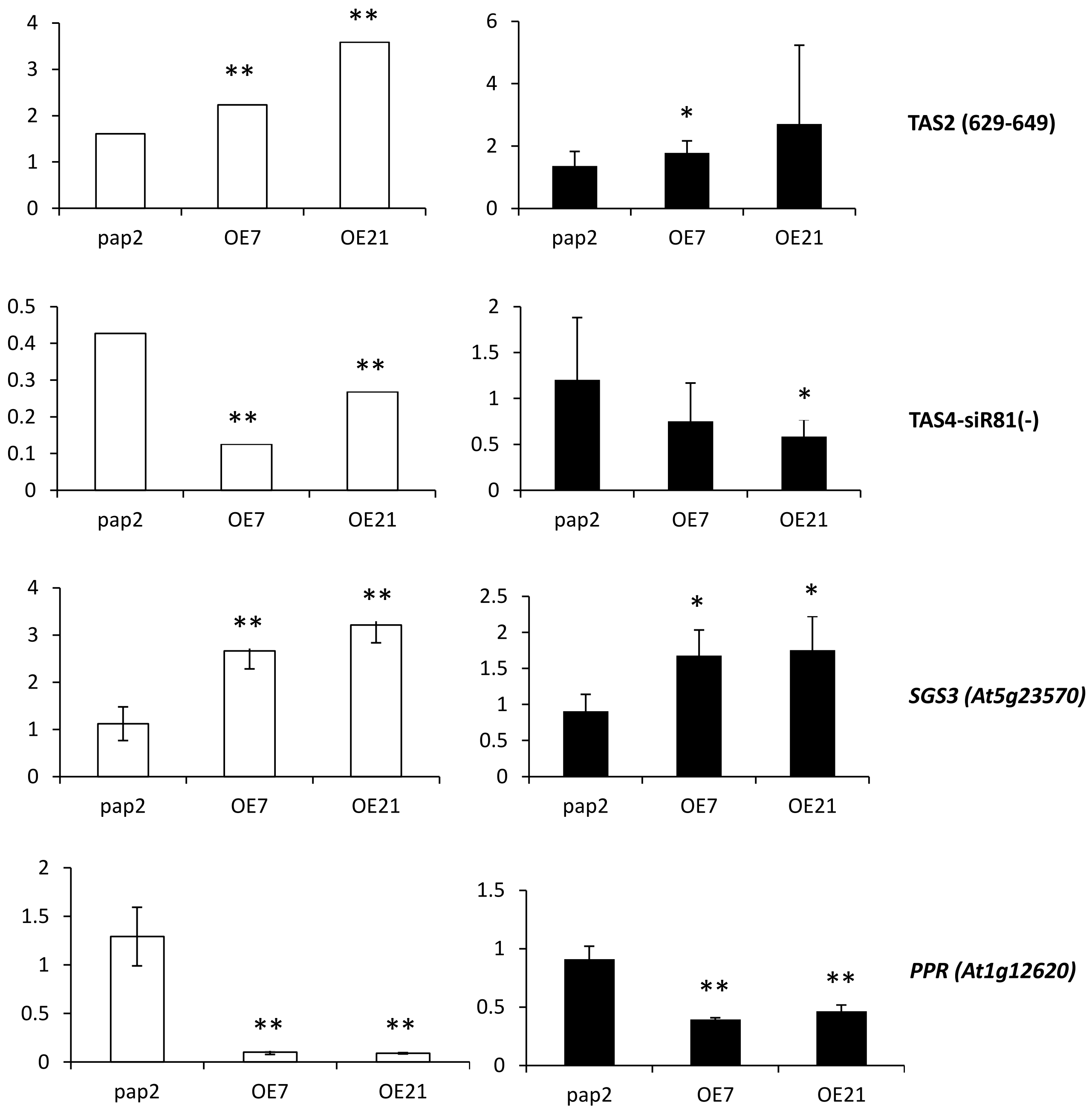

Additional file 15. Validation of small RNAs and microarray data by qRT-PCR. All the values are calculated by fold change (*pap2*/WT, OE7/WT or OE21/WT). Columns in white indicate small RNAs reads or microarray data, black columns indicate real-time RT-PCR data. Asterisks indicate significant difference compared with WT. * $P < 0.05$, ** $P<0.01$.
