# Supplementary material for: Global small RNA analysis in fast-growing Arabidopsis thaliana with elevated concentrations of ATP and sugars
Source: BMC Genomics. 2014 Feb 10;15:116. doi: 10.1186/1471-2164-15-116 (PMC3925372; doi:10.1186/1471-2164-15-116)
Supplement: Additional file 15 — Flowchart of small RNAs mapped to cis-NATs and trans-NATs in Arabidopsis by SOAP3. [file 1471-2164-15-116-S15.pdf]

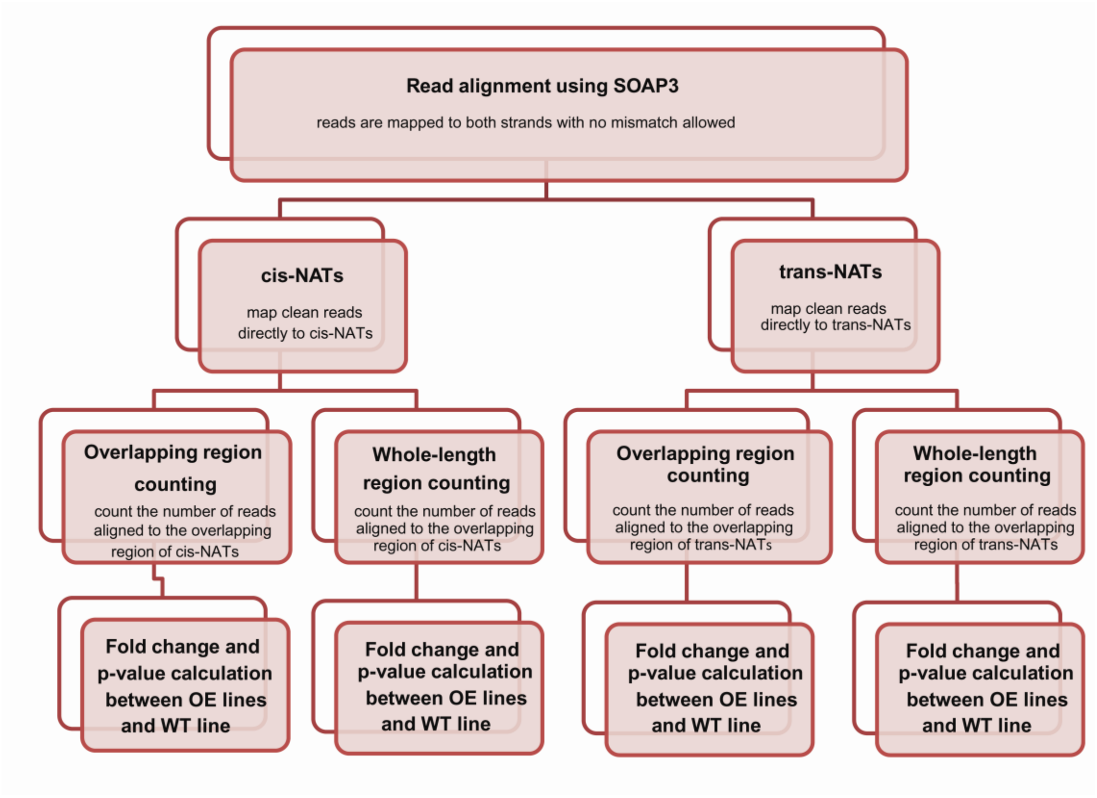

Additional file 14. Flowchart of small RNAs mapped to cis-NATs and trans-NATs in Arabidopsis by SOAP3.
